# Implementing STEADI for routine falls prevention of all older adults attending outpatient physical therapy: key partner perspectives

**DOI:** 10.3389/frhs.2025.1718490

**Published:** 2026-02-18

**Authors:** Jennifer L. Vincenzo, Mariana Wingood, Sarah K. Council, Aaron J. Scott, Jennifer S. Brach, Geoffrey M. Curran

**Affiliations:** 1 Department of Physical Therapy, University of Arkansas for Medical Sciences Northwest, Fayetteville, AR, United States; 2 Center for Implementation Research, University of Arkansas for Medical Sciences, Little Rock, AR, United States; 3 Department of Implementation Science, Wake Forest University School of Medicine, Winston-Salem, NC, United States; 4 Institute for Community Health Innovation, University of Arkansas for Medical Sciences Northwest, Springdale, AR, United States; 5 Department of Physical Therapy, University of Pittsburgh, Pittsburgh, PA, United States; 6 College of Pharmacy, University of Arkansas for Medical Sciences, Little Rock, AR, United States

**Keywords:** consolidated framework for implementation research, evidence-based practice, implementation science, injury prevention, mixed methods, rehabilitation

## Abstract

**Introduction:**

Falls are a leading cause of morbidity and mortality among older adults. Despite Prevention initiatives have been researched primarily in medical care. There is a dearth of studies on falls prevention in other primary care settings, such as outpatient physical therapy (PT). The purpose of this exploratory mixed methods study was to identify key partners' perceived barriers and facilitators to implementing the CDC's Stopping Elderly Accidents, Deaths, and Injuries (STEADI) falls-prevention initiative as routine care for older adults receiving outpatient PT.

**Materials and methods:**

Using a sequential explanatory mixed methods design, we collected surveys assessing familiarity, acceptability, appropriateness, and feasibility of STEADI and falls prevention, followed by semi-structured interviews to explore barriers and facilitators. Participants included physical therapy providers (*n* = 16), physicians (*n* = 5), administrative staff (*n* = 3), patients (*n* = 10), and care partners (*n* = 10). Quantitative data were summarized descriptively, and qualitative data were analyzed using rapid thematic analysis mapped to the Consolidated Framework for Implementation Research 2.0.

**Results:**

Participants found STEADI acceptable, appropriate, and feasible for integration into routine outpatient PT. Barriers among therapists included time constraints and limited familiarity with STEADI while interest in learning and implementing STEADI and perceived compatibility were facilitators. Physicians supported therapists but expressed concerns about scope of practice and challenges with communication jargon. Administrative staff were willing to assist patients with screening. Patients and care partners were receptive to STEADI but anticipated that some older adults might resist participation.

**Discussion:**

Findings support the potential feasibility of implementing STEADI in outpatient physical therapy. Addressing provider education, workflow, and interdisciplinary collaboration can address barriers. Aligning STEADI with therapists' scope of practice, improving interprofessional communication, and raising public awareness of physical therapists' role in falls prevention are critical for adoption. Our findings will inform co-development of implementation strategies to address barriers and capitalize on facilitators to support STEADI adoption in outpatient physical therapy.

## 1 Introduction

Falls are the primary cause of injuries among adults aged 65 and older (1). The resulting increase in healthcare expenditure for non-fatal falls is $80 billion annually in the United States (US) alone (2). Many falls are preventable through evidence-based screening, assessment, and targeted interventions to minimize risk (3, 4). The American Geriatrics Society and British Geriatrics Society (AGS/BGS) (5) and the World Falls Guidelines (6) advise that *all* adults aged 65 and older receive annual falls risk screenings and that those at-risk receive multifactorial risk assessment and tailored interventions. Guidelines emphasize the importance of healthcare providers, including physical therapists, in addressing falls-risk among older adults (6).

Historically, falls prevention initiatives such as the CDC's Stopping Elderly Accidents, Deaths, and Injuries (STEADI) primarily promote falls prevention conducted by physicians in primary care settings (7). However, barriers at the clinic and provider levels—such as managing multiple comorbidities, limited visit time, and insufficient follow-up—have hindered its adoption and sustainability (8–10). Due to these challenges, it is necessary to promote the adoption of falls prevention initiatives such as STEADI in other primary care settings, such as outpatient physical therapy. Physical therapists are well-suited to conduct falls prevention as primary care providers for older adults due to their extended one-on-one time and frequent follow-up with patients, compared to other primary care providers such as physicians, nurse practitioners, and physician assistants (11).

Studies support that therapists have effectively implemented STEADI in community-based settings (12, 13). Additionally, one health system demonstrated that physical therapists can successfully implement STEADI falls screening and physical function tests into routine outpatient physical therapy of older adults (14), but did not investigate full STEADI implementation. A recent study found that physical therapists' adoption and use of certain STEADI components aligns with their perceived complexity—low complexity corresponds with self-reported high use, while high complexity corresponds with low use (15). However, no studies have explored the barriers and facilitators to implementing STEADI and the perspectives of multiple key partners (e.g., physical therapists, referring physicians, patients, care partners, administrative staff) about STEADI's implementability. According to the World Falls Guidelines (6), it is necessary to explore and identify barriers, facilitators, and contextual factors across individual, clinical, and health system levels to support implementation of falls prevention initiatives such as STEADI.

The purpose of this study is to explore key partners' perceived barriers, facilitators, and contextual factors to implementing STEADI-based falls prevention as routine care for all older adults attending outpatient physical therapy, regardless of referral reason. By identifying these contextual determinants, we will gain insights into the different key partners' views on the barriers, facilitators, feasibility, acceptability, and appropriateness of falls prevention implementation efforts. The findings will be used to develop implementation strategies to support STEADI adoption into routine outpatient physical therapy care of all older adults.

## 2 Materials and methods

We utilized a mixed methods sequential, explanatory methodology. We first collected and analyzed individual's survey data and then conducted follow-up interviews to explain or expand upon our survey findings (16). Reporting guidelines for Mixed Methods Reporting in Rehabilitation & Health Sciences (MMR-RHS) were followed (17). Quantitative surveys were employed to identify each group's (e.g., therapists, patients) self-reported familiarity and perceptions about therapists' implementing falls prevention and STEADI (18). Following survey completion, nested semi-structured interviews were utilized to expand on and explain survey findings. The Consolidated Framework for Implementation Research (CFIR) 1.0 was utilized to design our qualitative interview guide and version 2.0, which was published after our interviews, was utilized for coding (19, 20). CFIR is widely used in implementation research to guide multi-level assessment of determinants of implementation and is explicitly recommended by the World Falls Guidelines to assess implementation context (6). Our study was approved by the University Institutional Review Board, IRB#274749.

### 2.1 Sampling, recruitment, eligibility, & consent

Purposive sampling was utilized to recruit key partners who may be involved in or impacted by STEADI implementation. We aimed to recruit the following key partners to participate in both surveys and interviews: all 20 *Physical Therapy Providers including two clinic managers (Therapists)* who worked full time in one of five participating clinics*,* all five *Administrative staff,* five *Physicians—*one per clinic—who referred older adult patients to the clinics*,* 10 older adult *Patients* who have had physical therapy in the past five years*,* and ten *Care Partners* of older adults who have had physical therapy in the past five years. A sample size of 5–10 per homogeneous group is recommended to gain data saturation in individual interviews (16). Recruitment, surveys, and interviews were conducted from October 2022 through June 2023. Consent was obtained electronically. Interviews were conducted and recorded via zoom and transcribed verbatim. Interviews lasted between 15 and 60 min.

#### 2.1.1 Internal key partners (clinics, therapists, clinic managers, and administrative staff working within the 5 outpatient physical therapy locations)

Clinics were invited to participate if they resided in the health system of interest, treated older adults, and the clinic management agreed to participate. Three clinics in the health system were not included in the study. One lacked resources due to involvement in a randomized control trial. Two others focused on sports rehabilitation and saw few older adults. Participants in this key partner group were *≥*18 years old, currently working at one of five targeted outpatient therapy clinics, and able to use a smart device (phone, tablet, or laptop) to complete the survey.

Study information was shared during regular clinic staff meetings, followed by an email with details and a survey link. Consent was obtained electronically. Eligible participants received up to three email reminders. Non-respondents were considered unavailable or uninterested. Managers were also practicing therapists; therefore, they were asked the same survey and interview questions as the staff therapists and those data were combined.

#### 2.1.2 External key partners (physicians, patients, and care partners)

Physicians self-reportedly treating ≥50% older adults in their caseload and referring to at least one of the five clinics were eligible for study participation. Therapists and management identified physicians, who were then emailed and invited to participate by the primary investigator.

Patients aged ≥65 who attended physical therapy in the past five years were eligible for study participation. Care partners were eligible if they were ≥18 and provided support (e.g., driving, cooking) to an older adult who had outpatient physical therapy in the same timeframe. Patients and care partners were not recruited as dyads. Recruitment methods included: (1) therapists shared study info and provided a paper survey or a flyer with a link to the electronic survey with interested participants, and (2) a statewide voluntary research registry.

Participants received either a paper-based survey or a survey link via a QR code or email, depending on preference. Eligible patients and care partners who completed the survey were invited to an interview, with up to three contact attempts. Non-respondents were considered unavailable or uninterested. The study team contacted eligible patients and care partners who completed the survey through the research registry, with recruitment focused on obtaining a representative sample of participants.

### 2.2 Data collection

Survey questions, distributed via Research Electronic Data Capture (REDCap), included sociodemographics and questions from Weiner et al.'s (18) validated surveys: Acceptability of Intervention Measure (AIM), Intervention Appropriateness Measure (IAM), and Feasibility of Intervention Measure (FIM), tailored to each key partner group (21, 22). Each survey contains 4 questions scored on a 5-point Likert scale. Mean scores of >3/5 indicate acceptability, appropriateness, and/or feasibility (18). Therapists and were asked about STEADI knowledge and falls prevention, STEADI use, and perceived complexity (0, not complex, to 10, complex). Physicians were asked about STEADI awareness and perceptions about therapists conducting different components of STEADI. Patients and care partners were asked about their knowledge and perceptions about falls/risks, patients' falls/risk, and perceptions of therapists' implementing different STEADI components. Administrative staff were queried on their perceptions about the STEADI screening questionnaire and helping older adults complete the questionnaire.

Qualitative interviews were conducted after surveys to expand or explain survey responses perceived barriers and facilitators to STEADI implementation. The semi-structured interview guide, based on CFIR 1.0 constructs and follow-up questions about survey responses, specifically identified barriers and facilitators, were reviewed by two physical therapists from other health systems who were familiar with STEADI, three external older adults and care partners, and one physician who were unfamiliar with STEADI. Feedback was used to improve the clarity of the interview guide to elucidate quantitative findings. All participants were educated on and shown the STEADI algorithm (Supplementary Figure S1) at the beginning of the interview.

#### 2.2.1 Remuneration

All participants received electronic gift cards after they completed both the survey and interview.

### 2.3 Data analysis

We first analyzed quantitative data followed by qualitative data. We then conducted an integrative analysis of quantitative findings with qualitative findings, examining areas of confirmation, expansion, or contradiction from one another to illuminate potential explanations (16).

#### 2.3.1 Quantitative data

We analyzed quantitative data using SAS/STAT version 9.4 (23). Means and standard deviations were calculated for continuous variables, and frequencies and percentages were calculated for dichotomous and categorical variables.

#### 2.3.2 Qualitative data

Our interviews were developed and conducted using CFIR 1.0 constructs, which was the only published CFIR available at the time. CFIR 2.0 was published after we conducted our interviews but before we started our data analyses. Therefore, we used the mapping guide in CFIR 2.0, to match the semi-structured interview questions, based on CFIR 1.0 constructs, to the coding template that was grounded in the updated and renamed constructs. This mapping technique ensures consistency between the interview guide, the coding template, and analysis (20). We performed a rapid thematic analysis of qualitative data using a pre-established coding template, developed from the domains and constructs of CFIR 2.0 (20, 24). CFIR 2.0 was released after our interviews, but before our data analysis. The qualitative analysis team was comprised of three qualitative researchers: JLV and MW, both board-certified geriatric physical therapists and implementation scientists, and SKC, a medical anthropologist. Each team member independently coded three transcripts before meeting to refine the coding template and resolve discrepancies through consensus. SKC then coded the remaining transcripts, with MW conducting a secondary review and JLV serving as an additional reviewer to address any inconsistencies. The team met weekly to address any discrepancies. Coded templates were then consolidated into a comprehensive matrix and refined through iterative team discussions. Illustrative quotes were selected for each theme and presented in the tables. The research team determined that they met data saturation, e.g., the point at which patterns in the data were clearly identified through analysis and no new themes emerged, after analysis of all transcripts (16).

#### 2.3.3 Mixed methods integration

We conducted an integrative analysis of quantitative and qualitative findings side by side to compare quantitative results with qualitative results, examining areas of confirmation, contradiction, and expansion.

### 2.4 Study participants

#### 2.4.1 Therapists (*n* = 16)

Sixteen of the eligible 20 therapists (80.0%) completed both the survey and interview. One completed part of the survey, and their partial data were included. Most therapists identified as female (56.3%), non-Hispanic White (93.8%), held a Clinical Doctorate (62.5%), had >6 years of experience (75.1%), and spent 76%–100% of their time in direct care (81.3%). Over 60.0% reported caseloads primarily of adults ≥65. See Table 1 for sociodemographic data.

**Table 1:** Descriptive summary—sociodemographic characteristics by group.

Measure	Category	Therapist/clinician (*n* = 16)	Referring physician (*n* = 5)	Admin. staff *(n* = 3)	Patients (*n* = 10)	Care partners (*n* = 10)
Age group						
	18–30	4 (25.0)	0 (0.0)	(0.0)	0 (0.0)	1 (10.0)
	31–40	5 (31.3)	2 (40.0)	(0.3)	0 (0.0)	0 (0.0)
	41–50	6 (37.5)	1 (20.0)	1 (33.3)	0 (0.0)	0 (0.0)
	51–60	0 (0.0)	0 (0.0)	1 (33.3)	0 (0.0)	2 (20.0)
	Over 60	1 (6.3)	2 (40.0)	1 (33.3)	10 (100.0)	7 (70.0)
	Unknown	0 (0.0)	0 (0.0)	0 (0.0)	0 (0.0)	0 (0.0)
Gender						
	Male	7 (43.8)	4 (80.0)	1 (33.3)	2 (20.0)	5 (50.0)
	Female	9 (56.3)	1 (20.0)	2 (66.7)	8 (80.0)	5 (50.0)
	Non-binary	0 (0.0)	0 (0.0)	0 (0.0)	0 (0.0)	0 (0.0)
Race and ethnicity						
	White (non-Hispanic)	15 (93.8)	4 (80.0)	1 (33.3)	7 (70.0)	8 (70.0)
	Black (non-Hispanic)	1 (6.3)	0 (0.0)	2 (66.7)	2 (20.0)	2 (20.0)
	AA (non-Hispanic)	0 (0.0)	1 (20.0)	0 (0.0)	0 (0.0)	0 (0.0)
	Hispanic	0 (0.0)	0 (0.0)	0 (0.0)	0 (0.0)	0 (0.0)
	NHPI (non-Hispanic)	0 (0.0)	0 (0.0)	0 (0.0)	0 (0.0)	0 (0.0)
	AIAN (non-Hispanic)	0 (0.0)	0 (0.0)	0 (0.0)	0 (0.0)	0 (0.0)
	Unknown	0 (0.0)	0 (0.0)	0 (0.0)	0 (0.0)	0 (0.0)
Marital status						
	Single (never married)	0 (0.0)	0 (0.0)	0 (0.0)	0 (0.0)	1 (10.0)
	Married/domestic partnership	0 (0.0)	0 (0.0)	0 (0.0)	7 (70.0)	8 (80.0)
	Widowed	0 (0.0)	0 (0.0)	0 (0.0)	1 (10.0)	0 (0.0)
	Divorced	0 (0.0)	0 (0.0)	0 (0.0)	2 (20.0)	1 (10.0)
	Unknown	16 (100.0)	5 (100.0)	3 (100.0)	0 (0.0)	0 (0.0)
Education						
	Less than High school degree	0 (0.0)	0 (0.0)	0 (0.0)	0 (0.0)	0 (0.0)
	High school degree or GED	0 (0.0)	0 (0.0)	1 (33.3)	0 (0.0)	1 (10.0)
	Some college, no degree	0 (0.0)	0 (0.0)	1 (33.3)	0 (0.0)	1 (10.0)
	Associate’s Degree	2 (12.5)	0 (0.0)	1 (33.3)	4 (40.0)	0 (0.0)
	Bachelor's Degree	1 (6.3)	0 (0.0)	0 (0.0)	1 (10.0)	3 (30.0)
	Master's Degree	2 (12.5)	0 (0.0)	0 (0.0)	2 (20.0)	3 (30.0)
	Professional Degree	0 (0.0)	0 (0.0)	0 (0.0)	2 (20.0)	2 (20.0)
	Clinical Doctorate Degree	10 (62.5)	0 (0.0)	0 (0.0)	1 (10.0)	0 (0.0)
	Doctorate Degree	0 (0.0)	1 (10.0)	0 (0.0)	0 (0.0)	0 (0.0)
	Doctor of Osteopathic Medicine degree (DO)	0 (0.0)	0 (0.0)	0 (0.0)	0 (0.0)	0 (0.0)
	Medical degree (MD)	0 (0.0)	5 (100.0)	0 (0.0)	0 (0.0)	0 (0.0)
	Unknown	1 (6.3)	0 (0.0)	0 (0.0)	0 (0.0)	0 (0.0)
Income						
	Less than $20,000	0 (0.0)	0 (0.0)	0 (0.0)	0 (0.0)	0 (0.0)
	$20,000 to $34,999	0 (0.0)	0 (0.0)	0 (0.0)	2 (20.0)	2 (20.0)
	$35,000 to $49,999	0 (0.0)	0 (0.0)	0 (0.0)	2 (20.0)	1 (10.0)
	$50,000 to $74,999	0 (0.0)	0 (0.0)	0 (0.0)	4 (40.0)	3 (30.0)
	$75,000 to $99,999	0 (0.0)	0 (0.0)	0 (0.0)	1 (10.0)	1 (10.0)
	$100,000–$149,000	0 (0.0)	0 (0.0)	0 (0.0)	0 (10.0)	1 (10.0)
	$150,000–199,000	0 (0.0)	0 (0.0)	0 (0.0)	0 (10.0)	1 (10.0)
	$200,000+	0 (0.0)	0 (0.0)	0 (0.0)	0 (0.0)	0 (0.0)
	Prefer not to answer	0 (0.0)	0 (0.0)	0 (0.0)	1 (10.0)	1 (10.0)
	Unknown	16 (100.0)	5 (100.0)	3 (100.0)	0 (0.0)	0 (0.0)
Occupation						
	Physical Therapy	12 (75.0)	0 (0.0)			
	Physical Therapy (management)	0 (0.0)	0 (0.0)			
	Physical Therapy Assistant	3 (18.8)	0 (0.0)			
	Occupational Therapy (management)	1 (6.3)	0 (0.0)			
	Medical Doctor	0 (0.0)	5 (100.0)			
	Unknown	0 (0.0)	0 (0.0)			
Certifications						
	Neurology	1 (6.3)	0 (0.0)			
	Women's Health	1 (6.3)	0 (0.0)			
	Dry Needling	1 (6.3)	0 (0.0)			
	No board certifications	9 (56.3)	0 (0.0)			
	Psychiatry and Neurology	0 (0.0)	1 (20.0)			
	Family Medicine	0 (0.0)	2 (40.0)			
	Anesthesiology Pain Medicine	0 (0.0)	1 (20.0)			
	Geriatric Medicine	0 (0.0)	1 (20.0)			
	Unknown	4 (25.0)	0 (0.0)			
Years in practice						
	<1 year	0 (0.0)	0 (0.0)			
	1 year	3 (18.8)	0 (0.0)			
	2–5 years	4 (25.0)	1 (20.0)			
	6–10 years	3 (18.8)	2 (40.0)			
	11–15 years	5 (31.3)	0 (0.0)			
	16–20 years	0 (0.0)	0 (0.0)			
	>20 years	4 (25.0)	2 (40.0)			
	Unknown	1 (6.3)	0 (0.0)			
Direct patient care (% of time)						
	76–100	13 (81.3)	3 (60.0)			
	51–75	1 (6.3)	1(20.0)			
	26–50	0(0.0)	1(20.0)			
	1–25	2(12.5)	0(0.0)			
	Unknown	0(0.0)	0(0.0)			
Percent of caseload age 65+						
	76–100	4(25.0)	2(40.0)			
	51–75	6(37.5)	2(40.0)			
	26–50	3(18.8)	1(20.0)			
	1–25	3(18.8)	0(0.0)			
	Unknown	0(0.0)	0(0.0)			

Percentages may sum to >100 due to rounding, or if multiple selections were available.

#### 2.4.2 Physicians (*n* = 5)

Seven physicians were invited and agreed to participate, but two did not complete the survey before the recruitment goal was met (one physician per clinic, *n* = 5, 100%) and were informed their participation was no longer needed.

The majority of physicians identified as male (80.0%) and non-Hispanic White (80.0%). All had 6 + years of clinical experience (80.0%) and spent 76%–100.0% of their time in direct patient care (60.0%). Just under two-thirds (62.5%) reported that 51–100.0% of their caseload was adults ≥65. See Table 1 for sociodemographic data.

#### 2.4.3 Administrative staff (*n* = 3)

Out of the five administrative staff in the clinics, three completed the survey (60.0%), and one completed the interview (20%). Ages were evenly split across 41–50, 51–60, and over 60. Most identified as female (66.7%) and non-Hispanic Black (66.7%). Education levels included high school, some college, and an associate's degree. See Table 1 for sociodemographic data.

#### 2.4.4 Patients (*n* = 10)

Eleven patients who were provided study information by therapists completed the survey, and eight agreed to be contacted for an interview. Two of those patients did not respond after three email attempts. Therefore, six of the eleven patients recommended by the therapists completed both the survey and interviews. To meet recruitment goals, the Arkansas Research Registry was used. Surveys were sent to adults ≥65 years of age. One hundred and thirteen older adults completed the survey. Older adults who agreed to be contacted for an interview were contacted via email until the desired sample size was achieved (*n* = 10). Six older adults were contacted and four interviews completed from the research registry. Data for the 10 patients who completed both the survey and interview (100.0%) are presented.

Eighty percent of patients were female, 70.0% were non-Hispanic, and 60.0% had a bachelor's degree or higher. Refer to Table 1 for sociodemographic data. STEADI scores are based on the average Stay Independent Questionnaire (SIQ)—a 12-question self-assessment for older adults. The average SIQ score was 7.9 (4.0), indicating increased falls risk. Eighty percent of patients considered themselves to be at risk of falling, and 60.0% had fallen in the past year.

#### 2.4.5 Care partners (*n* = 10)

Nine care partners who were provided study information by therapists completed the survey, and four agreed to and completed the interview. To meet interview recruitment goals, the Arkansas Research Registry was used. Surveys were sent to adults ≥18 years of age. From the research registry, 47 participants who self-reported as care partners of an older adult completed the survey, and six of nine care partners who agreed to be contacted completed the interview to reach our total target recruitment of 10 care partners. Data for the 10 care partners who completed both the survey and interview (100.0%) are presented.

Most care partners were over age 60 (70.0%), non-Hispanic White (70.0%), had a college degree (80.0%), and half were female (50.0%). Refer to Table 1 for sociodemographic data. Eighty percent of care partners reported that the older adult they assisted was at risk of falling, 60.0% reported that the older adult had a fall in the past year, and the average SIQ score of the older adult was 8.3(4.6), indicating an increased falls risk for the older adult the care partner assisted.

## 3 Results

Quantitative survey results are presented in the first section by each key partner group. In the second section, qualitative interview results are presented and integrated with quantitative survey findings.

### 3.1 Survey results

#### 3.1.1 Therapists

##### 3.1.1.1 Familiarity, use, and perceptions about implementing STEADI

All therapists felt that falls are not inevitable with age and can be prevented. Only 50.0% of therapists were somewhat familiar with the STEADI initiative; one participant was very familiar, 43.8% were not familiar, and 87.5% did not use STEADI. Table 2 delineates therapists' use of components that already align with STEADI in current clinical practice. For falls risk screening, most therapists reported using the Lower Extremity Functional Scale (68.8%) and the Activities Specific Balance Confidence Scale (62.5%) while 19 other falls risk tests/measures were used less than 31.3% of the time (Supplementary Table S1). For balance and functional falls risk assessment, 81.3% of therapists already reported using the timed up and go (TUG) and 30-second chair stand (30CS), and 31.3% used the 4-stage balance test, which are recommended in the STEADI (Table 2). Therapists also reported frequently using the Berg Balance Scale 75.0% of the time and 5-times sit to stand test 62.5% of the time to assess for falls risk (Supplementary Table S3).

**Table 2:** Therapists' reported use of STEADI components.

Components	Reported use *n* (%)
Screening questionnaire	1 (6.3)
Physical screening tests	
Timed up and go test	13 (81.3)
30-s chair stand test	13 (81.3)
4 stage balance test	5 (31.3)
None of these	0 (0.0)
Assessments/additional screens	
Foot problems and footwear	13 (81.3)
Orthostatic hypertension	13 (81.3)
Home safety	12 (75.0)
Comorbidities (e.g., depression, osteoporosis)	9 (56.3)
Cognition function^a^	9 (56.3)
Medications that increase falls risk	8 (50.0)
Vestibular function^a^	7 (43.8)
Urinary incontinence	5 (31.3)
Vision screen/encourage yearly check	3 (18.8)
Vitamin D intake	2 (12.5)
Interventions	
Strength training	16 (100.0)
Balance training	15 (93.8)
Gait training	15 (93.8)
Endurance training	15 (93.8)
Activity modifications for safety	12 (75.0)
Activities of daily living training	7 (43.8)
Floor transfer training^a^	7 (43.8)
Vestibular rehabilitation^a^	5 (31.3)
Educational Interventions	
Activity modifications/recommendations	12 (75.0)
Home safety recommendations	12 (75.0)
Family/care partners education	11 (68.8)
Footwear modifications and/or recommendations	11 (68.8)
Referrals to other healthcare providers to address modifiable risk factors not in PT scope of practice	9(56.3)

^a^ Falls risk components not in STEADI.

Therapists reported conducting some falls risk-related assessment STEADI related components. The majority reported assessing foot problems/footwear and orthostatic hypotension (81.3%) and home safety (75.0%). They less frequently reported assessing medications that increase falls risk (50.0%), urinary incontinence (31.3%), vision (18.8%), and vitamin D intake (12.5%; Table 2).

Therapists reported employing several fall-risk interventions that aligned with STEADI components (Table 2). All performed strength training, 93.8% performed balance, gait, and endurance training, and 75.0% provided activity modifications for safety. Less frequently performed interventions included activities of daily living training and non-STEADI vestibular rehabilitation (31.3%). Most therapists reported conducting the listed educational interventions; 75.0% for activity modification and home safety recommendations, 68.8% for family/care partner education and footwear recommendations, and 56.3% referred patients to other healthcare providers for modifiable risk factors outside the therapists' scope of practice.

Therapists found STEADI acceptable [AIM 4.5 (0.5)], appropriate [IAM 4.6 (0.6)], and feasible [FIM 4.5 (0.6)]. They reported assessing modifiable risk factors as the least acceptable [4.3(0.6)] and feasible [4.3(0.8)] STEADI components (Table 3). On average, therapists rated their confidence in conducting the STEADI screening, assessment, and interventions as 5.6/10 with 10 being the most confident. Over 87.0% cited time and 68.8% cited complexity as limiting factors for STEADI use. Regarding conducting specific STEADI components, the majority of therapists reported that time constraints were the greatest barrier associated with screening (50.0%), conducting physical tests (62.5%), assessing risks (93.8%), providing interventions (75.0%), and would hinder use (Supplementary Table S3).

**Table 3:** Therapist, physician, administrative staff, patients & care partners—perceptions of acceptability, appropriateness, and feasibility of STEADI being implemented in outpatient physical therapy.

Acceptability of Intervention Measure (AIM)					
Responses	Meets my approval	Is appealing to me	I like it	Is welcomed	Overall Mean (SD)
Respondent Group	M(SD)				
Referring Physician	4.6 (0.6)	4.4 (0.6)	4.6 (0.6)	4.6 (0.6)	4.6 (0.5)
Administrative Staff	4.3 (0.6)	4.3 (0.6)	4.7 (0.6)	4.3 (0.6)	4.4 (0.5)
Patients	4.6 (0.5)	4.4 (0.7)	4.4 (0.7)	4.5 (0.5)	4.8 (0.6)
Care partners	4.5 (0.7)	4.6 (0.5)	4.6 (0.5)	4.5 (0.5)	4.6 (0.5)
Therapists					
* Specific STEADI components*					
* *Screening questionnaire	4.6 (0.5)	4.6 (0.5)	4.6 (0.5)	4.5 (0.6)	4.6 (0.5)
* *TUG, 30CS and/or 4 stage balance test	4.5 (0.5)	4.3 (0.8)	4.3 (0.7)	4.4 (0.7)	4.4 (0.6)
* *Assessing modifiable risk factors	4.7 (0.5)	4.4 (0.6)	3.8 (1.0)	4.4 (0.8)	4.3 (0.6)
* *Fall risk interventions	4.8 (0.4)	4.8 (0.5)	4.7 (0.6)	4.7 (0.6)	4.7 (0.5)
Intervention Appropriateness Measure (IAM)					
Responses	Seems fitting therapy	Seems suitable	Seems applicable	Seems like a good match	Overall Mean (SD)
Respondent Group	M(SD)				
Referring Physician	4.6 (0.6)	4.6 (0.55)	4.5 (0.6)	4.5 (0.6)	4.6 (0.6)
Administrative Staff	4.7 (0.6)	4.7 (0.58)	4.7 (0.6)	4.7 (0.6)	4.7 (0.6)
Patients	4.6 (0.5)	4.6 (0.52)	4.5 (0.5)	4.5 (0.7)	4.6 (0.5)
Care partners	4.6 (0.5)	4.6 (0.52)	4.6 (0.5)	4.6 (0.7)	4.6 (0.5)
Therapists					
* Specific STEADI components*					
* *Screening questionnaire	4.4 (0.7)	4.5 (0.63)	4.6 (0.6)	4.5 (0.6)	4.5 (0.6)
* *TUG, 30CS and/or 4 stage balance test	4.5 (0.6)	4.5 (0.52)	4.6 (0.5)	4.4 (0.5)	4.5 (0.5)
* *Assessing modifiable risk factors	4.6 (0.5)	4.4 (1.02)	4.4 (0.8)	4.6 (0.6)	4.5 (0.7)
* *Fall risk interventions	4.8 (0.5)	4.7 (0.48)	4.8 (0. 5)	4.7 (0.5)	4.7 (0.5)
Feasibility of Intervention Measure (FIM)					
Responses	Seems implementable	Seems possible	Seems doable	Seems easy to use	Overall Mean (SD)
Respondent Group	M(SD)				
Referring Physician	4.6 (0.6)	4.6 (0.6)	4.6 (0.6)	4.6 (0.6)	4.6 (0.6)
Administrative Staff	4.0 (1.0)	4.7 (0.6)	4.0 (1.0)	4.3 (0.6)	4.3 (0.7)
Patients	4.3 (0.7)	4.4 (0.5)	4.3 (0.7)	4.3 (0.8)	4.3 (0.6)
Care partners	4.5 (0.7)	4.6 (0.5)	4.6 (0.5)	4.3 (0.8)	4.5 (0.6)
Therapists					
* Specific STEADI components*					
* *Screening questionnaire	4.6 (0.8)	4.6 (0.8)	4.6 (0.8)	4.7 (0.5)	4.6 (0.7)
* *TUG, 30CS and/or 4 stage balance test	4.6 (0.6)	4.6 (0.5)	4.6 (0.5)	4.6 (0.5)	4.6 (0.5)
* *Assessing modifiable risk factors	4.4 (1.0)	4.3 (1.0)	4.4 (0.8)	4.3 (0.6)	4.3 (0.8)
* *Fall risk interventions	4.6 (0.6)	4.5 (0.6)	4.6 (0.6)	4.6 (0.6)	4.6 (0.5)

TUG, timed up and go test; 30CS, 30 s chair stand test; M, Mean; SD, standard deviation.

Using the Stages of Change Model at the beginning and end of the survey (25), therapists were asked questions about their current use or intent to use STEADI in the next 6 months. Initially, 31.3% of respondents were in pre-contemplation with no intent to use STEADI, and 56.3% were in contemplation and considering using STEADI. At the end of the survey, only 6.3% of respondents were in pre-contemplation, and 75.0% were in contemplation (Supplementary Table S4). All therapists reported being somewhat to very likely to use all STEADI components with proper education.

#### 3.1.2 Physicians

##### 3.1.2.1 Familiarity, use, and perceptions about therapists implementing STEADI

All physicians felt that some falls are preventable and all but one felt that falls were not inevitable with aging. Only one of five physicians was familiar with STEADI but did not use it. Physicians perceived STEADI as acceptable [AIM 4.6(0.5)], appropriate [IAM 4.6 (0.6)], and feasible [FIM 4.6 (0.6)] for therapists to implement with older adults (Table 3). Physicians were asked about their perceptions of therapists implementing different components of STEADI. All STEADI screening, assessments, interventions, and referrals were supported by most physicians, with the exception of therapists' assessing urinary incontinence (40.0%), recommending supplemental vitamin D (40.0%), and managing orthostatic hypotension (20.0%; Supplementary Table S5). Physicians were confident (8.4/10) in therapists' using STEADI components they felt were within the therapists' scope of practice. Physicians also reported that it is moderately complex (5.4/10; 10 is complex) to interact with a therapist to address an older adult's modifiable risk factors for falls.

#### 3.1.3 Administrative staff

##### 3.1.3.1 Perceptions about administering STEADI questionnaire

Administrative staff were asked about their perceptions of administering the STEADI Stay Independent Questionnaire (SIQ) screening portion of STEADI to older adults. They reported it was acceptable [AIM 4.4 (0.5)], appropriate [IAM 4.7 (0.6)], and feasible [FIM 4.3 (0.7)] (Table 3). Administrative staff rated the appropriateness of assisting a patient to complete a questionnaire as 8.3(2.9) on a scale of 0–10 (0 not appropriate, 10 appropriate). Staff reported the feasibility as 8.7(2.3) and their confidence in assisting an older adult to complete the SIQ as 10.0(0.0).

#### 3.1.4 Patients

##### 3.1.4.1 Perceptions about STEADI

Patients perceived that it was acceptable [AIM 4.8(0.6)], appropriate [IAM 4.6 (0.5)], and feasible [FIM 4.3 (0.6)] for therapists to implement STEADI as routine care with older adults regardless of the reason for referral (Table 3). Ninety percent felt it was acceptable for their therapist to ask them to complete the SIQ if a patient was being seen for a reason other than falls prevention. Over 80.0% of patients felt that STEADI falls risk assessments and interventions were acceptable. However, 30.0% of patients felt that therapists checking their blood pressure was not acceptable, and 40.0% felt that vision checks were not acceptable. One patient felt that none of the assessments were acceptable if unrelated to their reason for referral (Supplementary Table S6).

#### 3.1.5 Care partners

##### 3.1.5.1 Perceptions about STEADI

Care partners perceived that it was acceptable [AIM 4.6(0.5)], appropriate [IAM 4.6 (0.5)], and feasible [FIM 4.5 (0.6)] for therapists to conduct STEADI with an older adult regardless of the reason for referral (Table 3). All ten (100.0%) felt it was acceptable for therapists to ask about falls or have patients complete the SIQ, and over 80.0% felt the STEADI falls risk assessments and interventions were acceptable. Only 30.0% of care partners felt that therapists checking vision, recommending a patient see another healthcare provider for falls risk, or assessing or addressing medication or medical issues was not acceptable (Supplementary Table S7).

### 3.2 Qualitative data triangulated with quantitative data

**Figure 1:**
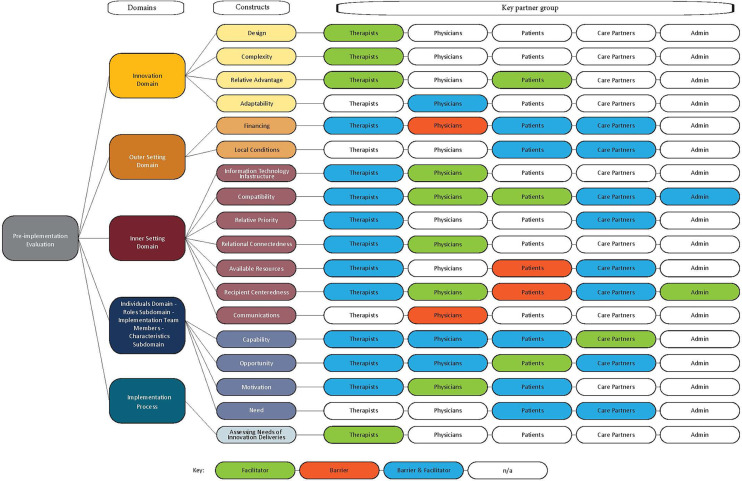
Key partner's barriers and facilitators to implementing STEADI mapped to constructs and domains of the consolidated framework for implementation research 2.0.

Results of qualitative analysis mapped to CFIR domains and constructs as a barrier, facilitator, or both, by each key partner group are presented in Figure 1. Qualitative data mapped to CFIR domains and constructs with exemplary quotes are reported in Tables 4–8. The most common domains, constructs, barriers, and facilitators are described below and, when possible, triangulated with quantitative findings. Note that not all interview-generated codes were represented in the survey items.

**Table 4:** Therapists perceived barriers and facilitators to STEADI implementation mapped to consolidated framework for implementation research 2.0 with exemplary quotes.

INNOVATION DOMAIN				
**Construct**	**Facilitator**	**Exemplary Quotes**	**Barrier**	**Exemplary Quote**
**Innovation Design** *The innovation is well designed and packaged, including how it is assembled, bundled, and presented.*	1. ***Therapists*** think STEADI is comprehensive and well-organized.	1. I think it's all-encompassing. I like it. I like the fact that it does refer out if necessary … I like it better than just a quick questionnaire screening tool and just, you know, being done. (16)	X	X
**Innovation Complexity** *The innovation is complicated, which may be reflected by its scope and/or the nature and number of connections and steps*.	1. ***Therapists*** think that STEADI is not complex.	1. Complexity I don't feel like is ever really as big of an issue if you have guidelines to follow, um, and give a clear and concise path in which to get from point A to point B to the end of whatever it is… I don't think is over the top by any means to get this implemented. (16)	X	X
**Relative Advantage** *The innovation is better than other available innovations or current practice.*	1. ***Therapists*** think STEADI (questionnaire, assessment, and interventions) is more comprehensive and better than what they currently do.	1. I think it's a good tool. It encompasses a lot of things that I would do anyways. I think that it covers a little bit more of the things that I don't necessarily think about, especially referring to podiatry, for example, is one of the things that I don't often talk to them about… for the most part, it's all-encompassing. (23)	X	X
OUTER SETTING DOMAIN				
**Financing** *Funding from external entities (e.g., grants, reimbursement) is available to implement and/or deliver the innovation.*	1. Implementing STEADI can lead to downstream revenue.	1. This has not only an impact on the patient, their safety, and those goals that we would have within our culture. Even from a leadership standpoint, even has downstream revenue attached to it…so I think overall that this will, should go off without a hitch, because it just has so many spokes to the wheel, right? That all just lead to positive outcomes for all involved. So even if there's a small opportunity cost that it might slow you down a little bit, again, the downstream impacts of that really benefit taking the time to do it. (16)	1. ***Patient*** finances can be a barrier to treatments.	1. I think too financially is something like, obviously is with insurance. and things like sometimes patients just can't afford to come into therapy, and so like, even though they may need that help, or that guidance or more one on one like I have to be more hands-off and kind of give them more of an exercise program and just hope that they're able to follow that and just kind of follow up with them via phone or email, or something like that. if they're copay, or that that goes too high, or something like that. (14)
**Information Technology Infrastructure** *Technological systems for tele-communication, electronic documentation, and data storage, management, reporting, and analysis support functional performance of the Inner Setting.*	1. ***Physicians*** who refer to three clinics report ease of using Epic for interprovider messages.	1. A simple message. Just be like, hey, you know, we evaluated, we saw this, we'd like to do this, And I'd be like, Great, go for it. I think Epic is probably the more HIPPA compliant official route for us, for us at least. (61)	X	X
**Relative Priority** *Implementing and delivering the innovation is important compared to other initiatives*.	1. ***Therapists*** and managers feel that they can make implementing STEADI a priority.	1. I think if we as a clinic made it a priority, I think we would have good buy-in because of the types of patients we see in our clinic…I think everyone already sees the importance of it. (30)	1. ***Therapists*** are concerned about prioritizing STEADI over the reason a patient is going to therapy.	1. Just my only issue would be … time management, especially if they come in for something else. (29)
**Relational Connections** *There are high quality formal and informal relationships, networks, and teams within and across Inner Setting boundaries (e.g., structural, professional)*.	1. ***Therapists*** feel like physicians are responsive to their concerns about patients. 2. ***Physicians*** welcome communication about patients from therapists.	1. In my experience, [physicians] have been very receptive to at least do a review of their medications, especially if I've had someone that's had a new medication and they've had a change in status. That's usually [the case], the doctors are actually very receptive and appreciative. (22) 2. Most of the PTs who work with us more closely here, you know, that feedback back and forth is always beneficial and everybody has the patients care in mind. So I've never had any problems in suggesting something to someone or the reverse happening. (56)	1. ***Therapists*** feel that having to contact a physician about issues is a barrier.	1. It seems like it's so involved and maybe the time management … having to contact the doctors and having that interdisciplinary communication would be probably the biggest barrier. (29)
OUTER SETTING DOMAIN				
**Construct**	**Facilitator**	**Exemplary Quotes**	**Barrier**	**Exemplary Quote**
**Available Resources** *Resources are available to implement and deliver the innovation.*	1. Two clinics have 60-minute time blocks for patient evaluations and treatments and feel they have adequate time.	1. We have one hour for all of our treatments (and) evaluations… So, depending on the time it takes to get the history from the patient as well as complete all of the other steps and assessments that we do … We'd be assessing for the primary reason for their visit first. This [STEADI] would be kind of a secondary assessment… we can do it in the follow-up visit. (30)	1. Three clinics have 30–45-minute time blocks and feel this may impact their ability to implement STEADI. 2. ***Therapists*** are concerned about the time to implement STEADI. 3. ***Therapists*** have concerns about patient resources to engage in fall prevention (financial or ability to access resources).	1. It could be a little bit time consuming, you know, whether they're 15 min late and we only get 45 min per eval. So, it's just how the clinic is set up. It's not really optimal for being really able to do due diligence … so I wish I got a bit more. (29) 2. You know my main concern is just the amount of time it will take to address each one of those things. (14) 3. I feel like that can definitely be a burden or affect outcomes … just the ability to access those resources. (14)
**Recipient-Centeredness** *There are shared values, beliefs, and norms around caring, supporting, and addressing the needs and welfare of recipients.*	1. ***Therapists*** think that fall prevention is part of providing holistic care for older adults. 2. ***Physicians*** feel that all providers have the patient's best interest in mind. 3. ***Physicians*** think that older adults have a need for fall prevention.	1. The benefits are just providing more holistic care for all of our patients, whether they're coming in for falls or not. And then the other benefit is in working in that way with patients leads to them saying, Hey, this group, this clinic took really good care of me. I highly recommend them for other friends.(30) 2. I mean it is all a team effort trying to take care of the patients… everybody has the patients care in mind. (56) 3. I think that could be a good idea. I mean, I think it's always I feel like sometimes fall risk gets overlooked. And so I could see. So anyway, that is that can fill the gaps is good. (64)	1. ***Therapists*** have concerns about patient buy-in.	1. Something that I could see happening is like a patient comes in for something totally unrelated and like, Why am I being given this questionnaire about falls? Maybe some of my more burly men that don't believe that there anything's ever going to happen to them or something, you know. Yeah, Even my women too. Same thing. and so I could see a few people, probably not very many, maybe taking it the wrong way as like. Oh, you're just giving this to me because I'm of age or something like that. (20)
**Communications** *There are high quality formal and informal information sharing practices within and across Inner Setting boundaries (e.g., structural, professional).*			1. ***Physicians*** have challenges understanding therapists’ communications.	1. Well, probably more on my side is I don't understand physical therapy as much as you guys do… I notice that sometimes they tend to use abbreviations that I'm not familiar with, it's like learning a different language when you're trying to communicate with somebody. Occasionally that leaves me scratching my head wondering exactly what that means… It's hard to interpret what they're telling me in this report. I think occasionally that may happen for any discipline, but I think the physical therapy, you guys use a lot of acronyms and abbreviations and things that I probably am not as familiar with. If anything, you send a report, don't assume that physicians understand all of these abbreviations and acronyms. (57)
INDIVIDUALS DOMAIN – CHARACTERISTICS SUBDOMAIN				
**Construct**	**Facilitator**	**Exemplary Quotes**	**Barrier**	**Exemplary Quote**
**Roles - Implementation Team Members** *Individuals who collaborate with and support the Implementation Leads to implement the innovation, ideally including Innovation Deliverers and Recipients*. **Capability** *The individual(s) has interpersonal competence, knowledge, and skills to fulfill Role.*	1. ***Therapists*** are knowledgeable about many risk factors for falls. 2. ***Therapists*** are already using common falls risk screening tools. 3. ***Therapists*** report they are already doing comprehensive falls risk assessment if they have time, and it is related to their referral. 4. ***Therapists*** report they are using common PT-related interventions for older adults at-risk for falls. 5. ***Therapists*** feel that they are already implementing some components of STEADI in PT. 6. ***Physicians*** are comfortable with therapists assessing medications and contacting them about medications which increase falls risk or cause orthostatic hypotension. 7. ***Physicians*** are comfortable with therapists assessing orthostatic hypotension and providing non-pharmacologic interventions for orthostatic hypotension.	1. So from my experience, of course, if there is some kind of preexisting condition, their rate of falls increases, especially if whatever that condition is, has impaired their balance like a stroke or hip replacement, knee replacement, those kinds of things…strength and balance, cognitive impairment… (21) 2. ABC, TUG, SOT, 4 Stage Balance Test, FGA, 6 m Gait Velocity, 2MWT/6MWT, 5x sit to stand, 30 s sit to stand. (23) 3. Assess their strength in their lower extremities. And I'll do a few different balance tests as well… look at their gait. I'm gonna look at their strength and all the major muscle groups of their lower extremities.. take a quick look at their medications. But if nothing is new, then I you know, kind of rush over that. I'll do the timed up and go typically. I'll test different positions like how long can they stand in narrow base of support, tandem, single leg, stance stuff like that … as far as their balance, I'll ask them about vertigo most of the time. See if they have any inner ear issues, and then … sometimes I'll ask about their vision, too, if they're wearing glasses or something… but usually that's in the chart, you know. Like if they have like blindness or something like that. (14) 4. Strength, interventions, doing things to improve people's mobility….balance exercises.. education making them more aware, changing things in their home environment. (27) 5. I feel it's a lot of things we do already in physical therapy, especially as a general practitioner. This doesn't work with one particular specialty. They're doing a lot of those things already with patients, or they're considering a lot of those already with patients. I don't think it looks hard to use … or it looks like oh, I've got something new I've never seen before.. I think we do a lot of that already. It's just a matter of … implementing it. It just isn't complicated. (27) 6. I'm all for you know, if y'all notice something that is known to cause an increase, far risk, send it to me. And if it's appropriate, I'll try to change it. (61) 7. Well, I assume you all are trained to take blood pressures appropriately, so I don't believe that would be a problem…, I would appreciate having that information because I obviously don't know everything that's going on with my patients. In terms of medication management other than stating to the patient that there might be an issue they need to discuss with their doctor, but in terms of some of the other things that you mentioned about the foot pumping, getting up slow and hold on, those are all appropriate for you to be able to visit with patients about. (57)	1. ***Therapists*** do not routinely screen older adults for falls. 2. ***Therapists*** do not mention assessments or interventions for orthostatic hypotension, feet/footwear, medication (physician referral) unless specifically asked about it. 3. ***Therapists*** are not knowledgeable or confident in using STEADI. 4. ***Therapists*** lack knowledge and/or skills for medication assessment. 5. ***Therapists*** report that it is out of their scope of practice to change an older adults’ medication. 6. ***Physicians*** are unsure about therapists training.	1. Some of our intake paperwork does not include any screening questionnaires for falls currently. Now there are specific ones. We can go ahead and might give a patient, but in terms of our new patient email paperwork it doesn't necessarily say, you know have have you fallen, or you know any specific questions to that. (17) 2. x 3. I just really am not familiar with that whole algorithm thing. (14) 4. The ones I'm probably lacking on, would be like the vitamin D or some medication and stuff like that. again. That's just not something I feel like I’ve probably done a lot of continuing ed on, and I need to kind of beef up a little bit. which I don't know why that is, I mean, we do pharm in school, but I think it's just like I'm not eager to go learn about medications, so I'd rather learn something that's more directly PT-related. I guess, even though I know medications are very important. But it's just like I guess it maybe is not as interesting for me to spend my time on outside of work or something like that. (20) 5. Medicine issues can cause dizziness, lightheadedness, and can be a contributor to falls, etc. If I see it's an ongoing problem, I just call the doctor and let them know. I said, you know, I'm worried about their stability. Could this be an issue, this medication? Well, you know, you don't want to step on the doctor's toes, and I don't want to be practicing outside of my practice act. …I'm not supposed to be making medication suggestions or lack thereof. (21) 6. And it's just it goes back to me being ignorant of your training. I don't know. Are you all trained to, you know, do orthostatic checks? (61)

**Table 5:** Referring physicians perceived barriers and facilitators to STEADI implementation mapped to consolidated framework for implementation research 2.0 with exemplary quotes .

INNOVATION DOMAIN				
**Construct**	**Facilitator**	**Exemplary quotes**	**Barrier**	**Exemplary quotes**
**Innovation Adaptability** *The innovation can be modified, tailored, or refined to fit local context or needs.*	1. ***Physicians*** feel that STEADI is appropriate for therapists to implement as long as therapists only conduct the components that are in their scope of practice.	1. In terms of the content, I don't really think so. The content looks pretty good to me… It's more just around making sure PT is practiced within their scope of practice. As far as when it says to stop, switch or reduce medications, that would be PT refer patient back to MD or something like that. (57)	1. ***Physicians*** feel that medication assessment and optimization should be deferred to the primary care or prescribing provider. 2. ***Physicians*** feel that comorbidities should be deferred to primary care.	1. The only concern I would have was with medication management, as in stopping switching or changing doses on medication, because you may not know the whole history on why someone is on a particular medication. I think it's important to give the education to the patient that these medications could cause a fall problem. And you want to talk to your prescribing physician about that particular medication. (56) 2. There are layers when it comes to identifying co-morbidities that I don't necessarily think physical therapists have the training to accurately say, you have this, this or this. (61)
OUTER SETTING DOMAIN				
**Financing** *Funding from external entities (e.g., grants, reimbursement) is available to implement and/or deliver the innovation.*	X	X	1. Home visits are not supported under the current financial/insurance infrastructure.	1. The same barriers we have. You know, you talk about home visits and I mean I mean, geriatric medicine, home visits have can be very, very valuable. But by the same token, they're not feasible. You know, you don't fit into the system we're in. It's like you can't see enough people in a day and you're not done. Reimbursement is not there its sad to say. Yeah that's but that's true. (44)
INNER SETTING DOMAIN				
**Information Technology Infrastructure** *Technological systems for tele-communication, electronic documentation, and data storage, management, reporting, and analysis support functional performance of the Inner Setting.*	1. Three clinics use Epic for interprovider messages.	1. A simple message. Just be like, hey, you know, we evaluated, we saw this, we'd like to do this, And I'd be like, Great, go for it. I think Epic is probably the more HIPPA compliant official route for us, for us at least. (61)	X	X
**Compatibility** *The innovation fits with workflows, systems, and processes.*	1. ***Physicians*** feel that PTs can implement STEADI because therapists see the patient more frequently. 2. ***Physicians*** feel that STEADI can be integrated into therapists’ workflow.	1. Yeah, you see ‘em much more often over a period of 6 to 10 weeks for therapy than I do, and they are only being seen once every 2 or 3 months. (57) 2. If it's filled out by them, it could be filled out in a waiting room on a. You know, waiting to go in for a visit to which would streamline things. (56)	X	X
**Relational Connections** *There are high quality formal and informal relationships, networks, and teams within and across Inner Setting boundaries (e.g., structural, professional).*	1. ***Physicians*** welcome communication about patients from therapists.	1. Most of the PTs who work with us more closely here, you know, that feedback back and forth is always beneficial and everybody has the patients care in mind. So I've never had any problems in suggesting something to someone or the reverse happening. (56)	X	X
INNER SETTING DOMAIN				
**Construct**	**Facilitator**	**Exemplary Quotes**	**Barrier**	**Exemplary Quote**
**Culture – Recipient-centeredness** *Shared values, beliefs, and norms around caring, supporting, and addressing the needs and welfare of recipients*.	1. ***Physicians*** feel that all providers have the patient's best interest in mind. 2. ***Physicians*** think that older adults have a need for fall prevention.	1. I mean it is all a team effort trying to take care of the patients… everybody has the patients care in mind. (56) 2. I think that could be a good idea. I mean, I think it's always I feel like sometimes fall risk gets overlooked. And so I could see. So anyway, that is that can fill the gaps is good. (64)	X	X
**Communications** *There are high quality formal and informal information sharing practices within and across Inner Setting boundaries (e.g., structural, professional).*	X	X	1. MDs have challenges understanding therapists’ communications.	1. Well, probably more on my side is I don't understand physical therapy as much as you guys do… I notice that sometimes they tend to use abbreviations that I'm not familiar with, it's like learning a different language when you're trying to communicate with somebody. Occasionally that leaves me scratching my head wondering exactly what that means… It's hard to interpret what they're telling me in this report. I think occasionally that may happen for any discipline, but I think the physical therapy, you guys use a lot of acronyms and abbreviations and things that I probably am not as familiar with. If anything, you send a report, don't assume that physicians understand all of these abbreviations and acronyms. (57)
INDIVIDUALS DOMAIN AND SUBDOMAINS				
**Roles - Implementation Team Members** *Individuals who collaborate with and support the Implementation Leads to implement the innovation, ideally including Innovation Deliverers and Recipients*. **Subdomain – Capability** *The individual(s) has interpersonal competence, knowledge, and skills to fulfill Role.*	1. ***Physicians*** are comfortable with therapists assessing medications and contacting them about medications which increase falls risk or cause orthostatic hypotension. 2. ***Physicians*** are comfortable with therapists assessing orthostatic hypotension and providing non-pharmacologic interventions for orthostatic hypotension.	1. I'm all for you know, if y'all notice something that is known to cause an increase, far risk, send it to me. And if it's appropriate, I'll try to change it. (61) 2. Well, I assume you all are trained to take blood pressures appropriately, so I don't believe that would be a problem…, I would appreciate having that information because I obviously don't know everything that's going on with my patients. In terms of medication management other than stating to the patient that there might be an issue they need to discuss with their doctor, but in terms of some of the other things that you mentioned about the foot pumping, getting up slow and hold on, those are all appropriate for you to be able to visit with patients about. (57)	1. ***Physicians*** are unsure about therapists training.	1. And it's just it goes back to me being ignorant of your training. I don't know. Are you all trained to, you know, do orthostatic checks? (61)
INDIVIDUALS DOMAIN AND SUBDOMAINS				
**Construct**	**Facilitator**	**Exemplary Quotes**	**Barrier**	**Exemplary Quote**
**Roles - Implementation Team Members** *Individuals who collaborate with and support the Implementation Leads to implement the innovation, ideally including Innovation Deliverers and Recipients*. **Subdomain - Opportunity** *The individual(s) has availability, scope, and power to fulfill Role.*	1. ***Physicians*** are comfortable with therapists implementing aspects of STEADI that are in therapists’ scope of practice	1. The content looks pretty good to me… It's more just around making sure PT is practicing within their scope of practice. (57)	1. ***Physicians*** are not comfortable with therapists changing medications. [NOTE- although this is part of STEADI algorithm, it is out of therapists’ scope of practice]. 2. ***Physicians*** are not comfortable with therapists assessing cognition.	1. The only concern I would have was with medication management, as in stopping switching or changing doses on medication, because you may not know the whole history on why someone is on a particular medication. I think it's important to give the education to the patient that these medications could cause a fall problem. And you want to talk to your prescribing physician about that particular medication. (56) 2. It's a little bit different from their typical scope [cognitive assessment]. Yeah. I may not be as comfortable with that. Maybe deferring that to the primary care doctor? (64)
**Subdomain - Motivation** *The individual(s) is committed to fulfilling Role.*	1. ***Physicians*** are motivated to support therapists to implement STEADI.	1. I'm all for physical therapy. And I absolutely if I want to be, I want to be notified if someone else notices something that could put the patient at risk. (61)	X	X

**Table 6:** Administrative staff perceived barriers and facilitators to STEADI implementation mapped to consolidated framework for implementation research 2.0 with exemplary quotes.

INNOVATION DOMAIN				
Construct	Facilitator	Exemplary quotes	Barrier	Exemplary quotes
**Compatibility** *The innovation fits with workflows, systems, and processes.*	STEADI is compatible with the clinic.	It's a great fit for our clinic to do the fall risk assessment (46).	***Patients*** may need help completing the questionnaire on paper.	We do get a few that need help (46).
**Recipient Centeredness** *The individual(s) has deficits related to survival, well-being, or personal fulfillment, which will be addressed by implementation and/or delivery of the innovation.*	There is a need to identify patients at risk of falls.	I feel like it's appropriate because the older patient has trouble walking and falls easier (55).	X	X

Domains that did not emerge in the data analyses are not represented in this table (Innovation, Outer Setting, Individuals, and Implementation Process).

**Table 7:** Patients perceived barriers and facilitators to STEADI implementation mapped to consolidated framework for implementation research 2.0 with exemplary quotes.

INNOVATION DOMAIN				
**Construct**	**Facilitators**	**Exemplary quotes**	**Barriers**	**Exemplary quotes**
**Relative Advantage** *The innovation is better than other available innovations or current practice.*	1. ***Patients*** feel that STEADI is a good tool and should be implemented.	1. Well, I think it's an excellent tool and it should be implemented… I mean, it's a good assessment tool. Bottom line. (37)	X	X
OUTER SETTING				
**Financing** *Funding from external entities (e.g., grants, reimbursement) is available to implement and/or deliver the innovation.*	1. The ***Patient*** is already attending physical therapy and should not incur additional costs.	1. I think if I’m going to travel to go to a therapist to get some assistance…all of them can ask whatever questions they need. (98)	1. There are financial barriers to implementing fall prevention.	1. I'm thinking with older adults, most of them are going to be on Social Security. I think the biggest barrier is financial for that for them. (37)
**Local Conditions** *Economic, environmental, political, and/or technological conditions enable the Outer Setting to support implementation and/or delivery of the innovation.*	1. The ***Patient*** is already attending physical therapy.	X	1. Some people may lack transportation.	1. Transportation becomes an issue for older people. (55)
INNER SETTING DOMAIN				
**Compatibility** *The innovation fits with workflows, systems, and processes.*	1. ***Patients*** feel that fall prevention can be incorporated into physical therapy.	1. Well, I believe it can be incorporated at the very beginning. On well, we just talked about the falls, the rugs. That could be implemented, the balance… I think it could be implemented at the beginning. I think, they can have printouts on falls. So, we'll have something in writing pertaining to adults and falling. (47)	X	X
**Available resources** *Resources are available to implement and deliver the innovation.*	X	X	1. Some ***Patient(s)*** may not have time, equipment, or someone to assist them with fall prevention at home.	1. Time, for instance, if the person is taking physical therapy and they're supposed to do so many things at home and they just don't have time or they don't think they need it or that's too much. (54)
**Recipient-Centeredness** *There are shared values, beliefs, and norms around caring, supporting, and addressing the needs and welfare of recipients.*	X	X	1. ***Patients*** feel this topic can be sensitive and clinicians need to be careful about how they engage older adults.	1. I would probably well in me, I would probably think it would be unnecessary, but if it's done in a way that is not I don't know inappropriate, or I know that would never be intended to make someone feel bad about their condition.. Somebody might be sensitive about their short fallings and that might just bring it to a point if they go there for a back. And somebody starts talking to them about falling. Well, that may. Okay, okay, so maybe delivering it in a sensitive manner. (170)
INNER SETTING DOMAIN				
**Construct**	**Facilitators**	**Exemplary quotes**	**Barriers**	**Exemplary quotes**
**Capability** *The individual(s) has interpersonal competence, knowledge, and skills to fulfill Role.*	1. ***Patients*** have some knowledge about falls risk and prevention factors.	1. I know they're very susceptible to and vulnerable to falls, mainly because of not taking care of things in their home like rugs and using a very hot polish on a floor or maybe a ceramic tile. And they get out of the shower, even using oils in the shower and slipping just from using things like that… I think most of them [falls] probably are preventable if we if we're conscious of it. (54)	1. ***Patients*** often mention being ‘careful’ which is not an evidence-based falls prevention intervention.	1. …falls are sometimes preventable by being careful. (26)
**Opportunity** *The individual(s) has availability, scope, and power to fulfill Role*.	1. ***Patients*** think they or others will be made aware of their falls risk and make modifications.	I see the therapist as being able to piece things together to maybe identify another thing or maybe something else that the patient's not aware of. (98)	X	X
**Motivation** *The individual(s) is committed to fulfilling Role*.	1. ***Patients*** feel that they would be open to interventions of prevent a fall.	I think anything that's preventative, you know, that might help, I wouldn't object. (270)	1. ***Patients*** think that some people may feel it is unnecessary or be insulted if fall prevention is included in physical therapy.	1. The challenge for the person accepting, it well, just some of those things that I mentioned, they might not feel like they need it. They can even be insulted that someone starts out on that, and they don't think they need it. (173)
**Need** *The individual(s) has deficits related to survival, well-being, or personal fulfillment, which will be addressed by implementation and/or delivery of the innovation.*	1. ***Patients*** think it is a good opportunity to include fall prevention in physical therapy.	1. I think it's a great opportunity because you're being proactive… I think a lot of folks assume because they're older, they take it for granted that they're going to have issues, not understanding that if they're proactive, they can possibly stay stronger. (37)	1. ***Patients*** are unsure of what age people may need to engage in fall prevention.	1. Somebody at 65 might not need to be reminded of some of it as somebody at 75….Some people are more agile at different ages. (173)

**Table 8:** Care partners perceived barriers and facilitators to STEADI implementation mapped to consolidated framework for implementation research 2.0 with exemplary quotes.

INNOVATION DOMAIN				
**Construct**	**Facilitators**	**Exemplary quotes**	**Barriers**	**Exemplary quotes**
**Not applicable**	X	X	X	X
OUTER SETTING				
**Financing** *Funding from external entities (e.g., grants, reimbursement) is available to implement and/or deliver the innovation.*	1. Preventing a fall is cost-effective. 2. The ***patient*** is already paying for therapy.	1. It could also save money because once that person falls, there are a lot of expenses that may end up on the taxpayers. So, it's like, an ounce of prevention is worth a pound of cure, that kind of thing. (238) 2. Money shouldn't be a factor because you are there. (36)	1. Money is a limiting factor.	When you’re older you don't have the funds to do some stuff and so money's always an issue. (48)
**Local Conditions** *Economic, environmental, political, and/or technological conditions enable the Outer Setting to support implementation and/or delivery of the innovation.*	1. The ***Patient*** is already attending physical therapy.	1. Well, if you are receiving physical therapy… you're already there. You've made it there. So transportation isn't the problem because you're there. (36)	1. Transportation and money are barriers to physical therapy.	1. Getting there and paying for it are the two biggest barriers. (238)
INNER SETTING DOMAIN				
**Compatibility** *The innovation fits with workflows, systems, and processes.*	1. ***Care partners*** feel that including fall prevention is compatible within the physical therapy visit.	1. Well, you are in that bracket where you could fall at that age. So it wouldn't be out of the ballpark not to, not to do that….it should go along with the rest of what you are being assessed for… I think anything that could be, beneficial to the older person should be included. (48)	1. Excess paperwork may be a barrier.	1. I don't know any patients who like to walk into a clinic and they've got a stack of paperwork that they're supposed to fill out, this looks like this and, and, you know, half the things they don't see the relevance of, and the other half are things that they've already been asked. And so people, people are not terribly happy about filling out paperwork. (49)
**Relative Priority** *Implementing and delivering the innovation is important compared to other initiatives.*	1. Fall prevention should be included in every session.	1. It should be done and talked about and almost part of every single session. (304)	1. Having other priorities for attending physical therapy may be a barrier.	1. You know, they may feel like I'm here. This is my priority. Why I'm here. Please take care of me for this. I'll look at your [fall prevention] paperwork later. (258)
**Available resources** *Resources are available to implement and deliver the innovation.*	1. There is adequate time in a physical therapy session to include fall prevention.	1. It could really be incorporated in. You know, you’ve got what a 50-minute session? I don't know. Some of ‘em are 50 min, and some of ‘em are an hour. But, um, you know, take five minutes of each session, and educate, communicate, roleplay… educate ‘em the other times that they’re sittin’ there takin’ a break. (304)	1. There is a concern if clinicians will have the time to provide the additional information about falls. 2. There is a concern about health literacy about fall prevention education materials.	1. I think there's so much information that could be provided, there might not be enough time and the providers might be constrained. (238) 2. She needs to have it written down so she can take it with her. She may not remember. She may misunderstand. She may just not get it… It needs to be written really easy to understand terminology if possible, because people have different levels of education and understanding. (238)
**Recipient-Centeredness** *There are shared values, beliefs, and norms around caring, supporting, and addressing the needs and welfare of recipients.*	1. ***Care partners*** suggest it is beneficial to make fall prevention personal and aligned with older adults’ priorities.	1. [Say] hey, we're going to make you feel, you feel better, your shoulder feel better. But if you fall, it's going to be vulnerable to being injured again and maybe connect it [fall prevention] to whatever they are there for right then, even though that's their priority. (238)	1. ***Care partners*** observe lack of adherence to home exercise programs. 2. ***Care partners*** fear that older adults may be offended or upset if they are told they are at risk of falling.	1. She has been given some home exercises that she's supposed to be doing, but she really doesn't do them very often. And, you know, other than that she tries to do what they were telling her, but she really isn't doing the exercises. (49) 2. The PT themselves… the person who's gonna be asking these things, making sure they are bringing this up in a way that isn't, like, accusing or, like, calling them a fall risk and stuff. Make sure they handle it with respect and poise (489)
INNER SETTING DOMAIN				
**Construct**	**Facilitator**	**Exemplary Quotes**	**Barrier**	**Exemplary Quote**
**Capability** *The individual(s) has interpersonal competence, knowledge, and skills to fulfill Role.*	1. ***Care partners*** are knowledgeable about what causes falls and how to prevent them.	1. Well, I know from firsthand experience it can be very detrimental and costly and hard on the patient and hard on the care partner. Uh, it's something that needs to be avoided at all possibilities. We try really hard…I can pull into the garage. He can get out of the car with his U-Step and go directly into the house. There are no steps… make the bathroom safe as it can be. we don't have carpeting, minimal throw rugs, in areas that he doesn't walk in…We try to be very cautious. (25)	X	X
**Opportunity** *The individual(s) has availability, scope, and power to fulfill Role*.	1. The ***Patient*** is already attending physical therapy.	1. The more she knows about how to help protect her and the more I know I think is great.. she's going for physical therapy. That is the opportunity for those health providers to educate her. (238)	1. Nobody treats the whole ***Patient***, they only focus on their niche.	1. Nobody takes the time to-to really treat the whole patient. Everybody focuses on the one little niche, and that's it. So, you know, you wonder why they’re at a fall risk. (304)
**Need** *Need - The individual(s) has deficits related to survival, well-being, or personal fulfillment, which will be addressed by implementation and/or delivery of the innovation*.	1. ***Care partners*** feel positively about fall prevention being included in physical therapy for older adults.	1. I think that would be good. Yes, because sometimes, you know, you notice things before them. You could actually say, Oh, I need to do something about that. And if that will kind of trigger more than awareness, for them that will probably help some of it… Excellent! Yes, that would be excellent. (258)	1. Denial of the ***Patient*** being at risk of falls is a concern.	1. Well, I can see a lot of people being resentful while I don't have that issue and I don't think I'm going to have that issue. So why are you wasting my time telling me something that's not going to impact me? That's still very prevalent in today's society. There's a lot of denial about a lot of issues. People just don't want to realize that they're getting older, that they maybe aren't able to do the things that they used to, or that they still want to because of, of limitations. So there, there's that negativity on the, the patient side of things. I think it also depends on, on who and how it's, it's being put to the client. Bedside manner plays a big role in how people perceive things or are willing to accept things. (36)

#### 3.2.1 Domain 1: innovation characteristics

Within the innovation characteristics domain, the majority of emergent codes were associated with the following constructs: innovation design, innovation complexity, relative advantage, and adaptability.

##### 3.2.1.1 Facilitator: design, complexity, and relative advantage for therapists

Therapists described STEADI as comprehensive, well-organized, and superior to other fall screenings, providing a clear path for addressing falls risks that are typically not addressed, i.e., referral to podiatry. Although 68.7% reported in surveys that complexity would somewhat to greatly hinder their use of STEADI, follow-up interview questions (which included the interviewer reporting back their survey answers) revealed discordance in that they did not actually view complexity as a barrier when reflecting on their responses.

##### 3.2.1.2 Facilitator: relative advantage for patients

Patients felt that STEADI is beneficial, a good tool, and should be implemented in falls prevention efforts. This aligned with the patient survey data that implementing STEADI is acceptable, appropriate, and feasible.

##### 3.2.1.3 Barrier and facilitator: physicians' concern of adaptability for therapists' scope of practice

Physicians felt it was appropriate for therapists to implement STEADI if limited to therapists' scope of practice. Interviews supported survey findings that physicians felt that STEADI components like assessing urinary incontinence, adding supplemental vitamin D, managing orthostasis, and modifying medications were not appropriate for therapists to address.

Physicians self-reported a lack of knowledge about therapists' scope of practice for some components of falls prevention, and three out of five asked the interviewer (JV) questions about the therapist's scope and expertise to assess and/or manage orthostatic hypotension, urinary incontinence, and cognitive function. Therapists' scope of practice for orthostatic hypotension is to evaluate if a patient is orthostatic, provide education on non-pharmacological management of orthostasis (i.e., educate the patient to do ankle pumps, get up slowly, hydrate), and refer the patient to the primary care physician for medical management of orthostasis. Therapists' scope of practice for high fall risk medications is to identify if a patient is on a fall risk increasing medication and refer the patient to their primary care physician if there is a concern. It is out of scope of practice for a therapist to prescribe, change, or discontinue medications (26). After JV educated them, physicians concluded that STEADI items outside of a physical therapist's scope of practice were medication assessment and optimization, and identification of comorbidities, which should be managed by primary care providers or the prescribing physician.

#### 3.2.2 Domain 2: outer setting

Within the outer setting domain, the identified codes were financing and local conditions.

##### 3.2.2.1 Barrier and facilitator: financing according to therapists, patients, and care partners

Therapists expressed concerns that financial constraints can be a barrier for patients attending therapy and, therefore, receiving STEADI. For patients, barriers to engagement in falls prevention included reliance on social security, fixed incomes, and the expenses associated with home modifications. Similarly, care partners worried that financial limitations significantly impact access to necessary interventions.

However, therapists emphasized that implementing STEADI can generate long-term revenue benefits. Patients contended that falls prevention measures should be included as part of their existing physical therapy without incurring extra costs. Meanwhile, care partners saw it as cost-effective in preventing future expenses from falls. There was no relevant quantitative data to report aligning with this theme.

##### 3.2.2.2 Barrier and facilitator: local conditions for patients and care partners

Transportation to physical therapy poses a challenge for some patients and is a significant barrier, according to care partners. However, both groups noted that patients are already attending physical therapy, which is a facilitator. The latter findings are consistent with survey findings: 90.0% of patients and 100.0% of care partners agreed that implementing STEADI while patients are already attending physical therapy would be beneficial.

#### 3.2.3 Domain 3: inner setting

Within the inner setting domain, the identified codes were information technology infrastructure, compatibility, relative priority, relational connectedness, available resources, recipient centeredness, and communications.

##### 3.2.3.1 Facilitator: compatibility for therapists, physicians, administrative staff, patients, and care partners

Therapists recommended incorporating STEADI into the workflow by including it in intake paperwork and the EHR for compatibility. Administrative staff felt that STEADI was a good fit for the clinic, and they could assist if an older adult needed help completing the SIQ. Physicians supported therapist-led implementation due to frequent patient contact, and the potential for STEADI being integrated into therapists' workflow. Patients and care partners felt that falls prevention could be incorporated into physical therapy visits. All key partners agreed that STEADI is feasible and 90.0% of patients and 100.0% of care partners agreed that it would be beneficial to implement STEADI while patients are already attending physical therapy.

##### 3.2.3.2 Barrier and facilitator: relative priority for therapists

Therapists and care partners were concerned about prioritizing STEADI at the expense of addressing the primary reason a patient is seeking therapy. However, both therapists and managers believed they could gain support and make the implementation of STEADI a priority. Survey data indicated that therapists perceived STEADI as feasible and appropriate to implement.

##### 3.2.3.3 Barrier and facilitator: available resources for therapists, patients, and care partners

Therapists and care partners were concerned whether clinicians would have time to address and engage patients in falls prevention, while some care partners felt there should be adequate time within the therapy visit to include falls prevention. Therapists who worked in two of the clinics that have 60-min time blocks for patient evaluations and treatments felt they should have adequate time. Conversely, therapists who worked in three clinics with 30–45 min time blocks for evaluations and treatments voiced concern that they may not have time to conduct STEADI. Survey data reinforces this concern from the therapists' perspective; 87.0% of therapists reported that time will somewhat to greatly impact their use of STEADI. Patients and care partners also reported concerns that patients may not have the time, equipment, or someone to assist them to engage in falls prevention at home.

##### 3.2.3.4 Barrier and facilitator: recipient centeredness for therapists, physicians, administrative staff, and care partners

Interview data revealed that the recipient-centeredness of identifying falls risk of an older adult receiving healthcare was a facilitator for all groups. Therapists had concerns about patient buy-in. Care partners echoed this fear and were concerned that older adults might be offended if they are told they are at risk of falling. Care partners emphasized the importance of therapists tailoring falls prevention strategies to align with the priorities of older adults. There was no relevant quantitative data to report that aligned with this theme.

##### 3.2.3.5 Barrier: communications for physicians

Physicians expressed concern over challenges in understanding therapists' communications due to therapists' use of acronyms and abbreviations. This aligns with the survey data that physicians reported the complexity of interacting with therapists (5.4/10). Despite the challenges, physicians rated their confidence in communicating with therapists as 8.4/10.

#### 3.2.4 Domain 4: individuals

Within the Individual domain, Roles subdomains of Implementation Team Members and Characteristics of Individuals, the identified codes were capability, opportunity, motivation, and need.

##### 3.2.4.1 Facilitator: capabilities for care partners

Care partners were knowledgeable about the causes of falls and strategies for prevention. In the survey data, 80.0% of care partners reported that the older adults they assisted were at risk of falling, and 60.0% indicated that these individuals had experienced a fall within the past year. Care partners reported a mean SIQ score of the older adults they care for as 8.3, reflecting an elevated falls risk of patients.

##### 3.2.4.2 Facilitator: opportunity for patients

Patients think they or others would be made aware of their falls risk and, therefore, make modifications to address their risks. This aligned with survey data; 90% of patients indicated that it's acceptable for a therapist to educate them on how to decrease their risk of falling.

##### 3.2.4.3 Barriers and facilitators: capability for therapists, physicians, and patients

Therapists were knowledgeable about the etiology of falls, risks, and reported already using common falls risk screening tools, including some assessments and intervention components within STEADI. However, not all therapists reported consistently using appropriate falls risk screening tools, and assessing risks like medications, urinary incontinence, and vitamin D intake. Survey data aligned with the interview data as far as therapists' use and familiarity with falls risk prevention. As implementation team members, physicians expressed uncertainty about therapists' capability, scope, and expertise in conducting different components of STEADI falls prevention but felt it was acceptable for therapists to assess medications (80.0%) and contact/refer physicians to communicate medications that increase falls risk or cause orthostatic hypotension (100.0%). Patients possessed some awareness of falls risks and prevention strategies but often report being “careful,” which is not an evidence-based intervention for falls prevention.

##### 3.2.4.4 Barrier and facilitators: opportunity for therapists

Therapists reported they commonly only address falls prevention if an older adult is referred to therapy for balance and/or falls. In the surveys, therapists reported that if they were provided education about STEADI that they would be likely to use it.

##### 3.2.4.5 Barrier and facilitators: motivation for therapists and patients

Therapists reported that they are willing to try implementing STEADI; however, some voiced concerns about coworkers' buy-in. Survey data indicated that therapists were in the contemplation or preparation stage to implement STEADI. Patients felt that older adults would be open to falls prevention as part of their therapy but voiced concerns that some people may feel it is unnecessary or be insulted if asked questions about falls and falls risk factors.

##### 3.2.4.6 Barrier and facilitators: need for patients and care partners

Patients were unsure of what age people may need to engage in falls prevention. Care partners were concerned that some patients were in denial about their falls risk. There was no relevant quantitative data to report for this theme.

#### 3.2.5 Domain 5: implementation process

Within the implementation process domain, the identified code was assessing needs of innovation of deliverers.

##### 3.2.5.1 Facilitator: assessing needs of innovation deliverers for therapists

Therapists feel that it is beneficial that the implementation team is assessing the needs of the therapists and other key partners that STEADI will impact.

## 4 Discussion

This study identified key partners' perspectives, within five clinics in an academic health system in the South, about contextual determinants that might impact the implementation and adoption of STEADI-based falls prevention in outpatient physical therapy. Research suggests that initiatives that integrate input from diverse key partners are more likely to address patient needs effectively and lead to sustainment and improvements in outcomes (27, 28). Overall, key partners perceived that STEADI is acceptable, appropriate, and feasible to implement in outpatient physical therapy for all older adults attending regardless of reason for referral. Barriers and facilitators that might impact adoption by each key partner group are discussed below.

### 4.1 Implementation barriers and facilitators supported by all groups

There were several common facilitators for implementing STEADI across all key partners. Consistently high ratings of acceptability, appropriateness, and feasibility, scores across therapists, physicians, administrative staff, patients, and care partners reflect the perception that STEADI is implementable. STEADI was perceived as comprehensive, valuable, and compatible with outpatient physical therapy. A systematic review on factors affecting implementation of evidence-based interventions in rehabilitation settings found that beliefs about an intervention, its relative advantage, and its compatibility with current practices serve as key facilitators (29). All key partners emphasized STEADI's patient-centeredness and the value of screening and addressing older adult falls risks. Research shows that settings that promote patient-centered care are more successful in implementing changes (20). The alignment of our results with these established facilitators further supports the feasibility of implementing STEADI in outpatient physical therapy.

Physicians, patients, and care partners felt that implementing STEADI during existing therapy visits reduces barriers to older adults engaging in falls prevention, such as transportation and costs. However, these access barriers likely still need to be assessed and addressed among older adults who would benefit from falls prevention and physical therapy, and should be considered in future investigations (10, 30). Falls prevention via home-based visits or remote therapeutic monitoring has been successful, yet financial barriers to access remain (31, 32).

### 4.2 Implementation determinants of therapists

Therapists' limited familiarity with STEADI explained their reported lack of use in clinical practice (29). After learning about STEADI through the surveys and interviews, therapists found it acceptable, appropriate, feasible, compatible, and not complex. Survey results supported that even increasing therapists' awareness of STEADI improved their consideration of utilizing it. A national survey (33) found high use among therapists who reported they were very familiar with STEADI. These data suggest that increasing therapists' awareness and familiarity with STEADI may be an appropriate implementation strategy to increase adoption (33). Education alone is usually insufficient to affect healthcare providers' adoption of evidence-based practices. Therefore, contextual determinants and tailored implementation strategies are also needed (34).

Though unfamiliar with the STEADI initiative, therapists reported that they frequently conducted many STEADI components that are already conducted commonly in physical therapy practice, such as gait, balance, and mobility assessments and interventions. Therapists less frequently assessed modifiable risk factors such as medications that increase falls risk, urinary incontinence, vision, and vitamin D intake. These findings are similar to another study where therapists in a different health system that implemented STEADI reported most frequently conducted gait, balance, and mobility components of STEADI and less frequently assessed medications and urinary incontinence (15). The study also found that therapists perceived STEADI components such as medication and urinary incontinence assessment as complex if they did not have the knowledge or confidence to conduct those components. Our findings with Kinney and colleagues' (29) findings that complexity and lack of knowledge are barriers to the adoption of evidence-based practices in rehabilitation. Some potential implementation strategies to address these barriers include educational interventions and clinical decision support (35). Integrating STEADI into clinical workflow and the EHR for successful implementation was recommended and reported as feasible by therapists in our study (14, 15), which have been strategies to support STEADI adoption in primary care (8, 10).

Time constraints and competing priorities, reported by 87.0% of therapists, were also barriers to implementing STEADI, and consistent with barriers to implementing STEADI and falls prevention in primary care (8, 9, 36, 37). The American Geriatrics Society recently recommended that STEADI be completed across multiple visits to support feasibility in primary care (38). Leveraging therapists' one-on-one time and frequent visits during an episode of care are important, as limited time and follow-up are barriers to implementation of evidence-based practices in rehabilitation (29). Furthermore, therapists who utilized STEADI in another health system reported that the ability to complete STEADI across multiple visits was a facilitator of adoption.

### 4.3 Implementation determinants of physicians

Physicians found therapist-led STEADI implementation acceptable, appropriate, and feasible, but were unclear about which components were within therapists' scope of practice. Implementation strategies to address physicians' awareness of therapists' professional roles and scope of practice can be facilitated through interprofessional collaboration and communication. However, physicians in our study reported that therapists' use of acronyms and jargon created communication barriers, which have also been identified in other studies as barriers to interprofessional practice (39, 40). Clear, standardized communication is a potential implementation strategy to support effective interdisciplinary communication, where physical therapists play a central role in implementing STEADI (6).

### 4.4 Implementation determinants of administrative staff

All staff found STEADI implementation in the clinic was acceptable, appropriate, and feasible, reporting that it would be beneficial to screen and treat older adults for falls risk. They also reported that some older adults may need assistance to complete the screening questionnaire, but that it should not be burdensome. A prior study investigating STEADI implementation in rehabilitation found that front desk staff providing the SIQ questionnaire via paper or iPad was a facilitator of implementation (15). This integral role and initiation of STEADI from the front desk staff is supported in recommendations for coordinating care across members of the health team to implement STEADI and falls prevention for older adults (41). It is also important to gain buy-in from all members of a healthcare team involved in implementing evidence-based falls prevention to support adoption and sustainability (42).

### 4.5 Implementation determinants of patients and care partners

Patients and care partners agreed that implementing STEADI as routine care in outpatient physical therapy was acceptable, appropriate, and feasible. A minority of patients and care partners had concerns about therapists checking their vision and blood pressure, mirroring physicians' uncertainty about therapists' scope to assess and manage orthostatic blood pressure. Prior research shows patients often lack awareness of physical therapy's role in fall prevention, highlighting the need to educate both providers and the public about therapists' role in falls prevention for older adults (43).

Though many older adults and care partners weren't fully aware of therapists' role in falls prevention, most supported its inclusion in therapy. Some care partners voiced concerns that falls and risks might be a sensitive topic for some older adults or not be able to be prioritized during therapy sessions, but older adults did not voice those same concerns. A recent scoping review found that older adults may feel embarrassed and concerned about losing their independence after a fall, or frequently did not perceive themselves to be at risk for falls (44). Literature-supported strategies that may address these issues are to ensure falls prevention recommendations are personalized to individual risks (45), provided by healthcare professionals (10), and engage shared decision-making. It is important to note for context that the care partners and older adults in our study both reported that the older adults were at risk of falling, and 60.0% had experienced a fall. Stevens and colleagues (46) found that older adults who have not fallen are less likely to acknowledge their risk and engage in falls prevention. Patient and care partner responses to and perceptions about falls prevention being integrated into therapy may be different among people who have not experienced a fall or are at a lower risk of falling.

Older adults and care partners in our study reported that a lack of access to resources such as equipment, home modifications, and exercise programs were barriers to older adults' engagement in falls prevention. Clinical community connections are necessary to improve older adults' knowledge and access to programs and available falls prevention interventions (47). Notably, engaging older adults in falls prevention, when included as routine care for older adults already attending outpatient physical therapy for any reason, overcomes many barriers to falls prevention engagement cited in the literature and reported as potential barriers by our respondents, such as insurance and costs, transportation, and access to physical therapy (30). However, these barriers still need to be overcome initially for at-risk older adults to have access to physical therapy and falls prevention (30, 46).

## 5 Conclusion

This study provides insights into the contextual determinants influencing the implementation of STEADI-based falls prevention in outpatient physical therapy. Across one health system, before it was implemented, STEADI was perceived by key partners as feasible, acceptable, and appropriate, reinforcing its potential to become a routine component of care for all older adults seen in outpatient physical therapy, regardless of referral reason. Despite high perceived feasibility, acceptability, and appropriateness ratings, barriers such as time constraints, therapists' training needs, and role-specific concerns must be addressed through implementation strategies to optimize STEADI adoption. There is also a critical need for the therapy profession to educate the public and other professionals about physical therapists' scope of practice in falls prevention, and for therapists to improve clarity in their communication with physicians.

Embedding STEADI falls prevention into routine outpatient physical therapy can address common implementation barriers found in primary care (e.g., patient access, engagement, transportation, and costs). However, patient-specific barriers to accessing equipment, home modifications, and community exercise programs still exist. Moreover, ensuring access to physical therapy and related interventions remains essential to fully leverage STEADI's potential to reduce falls among older adults. Understanding the views of key partners involved in STEADI implementation highlights each partners' unique challenges associated with adoption and identifies opportunities to tailor implementation strategies. This approach ensures that implementation efforts are contextually appropriate and positioned to improve the effectiveness of STEADI falls prevention efforts in outpatient physical therapy.

Study findings will inform next steps in co-developing tailored implementation strategies with key partners using approaches like evidence-based quality improvement (EBQI). EBQI fosters collaboration between implementation researchers and local partners to jointly identify and adapt strategies that fit the local context and support the adoption of evidence-based practices (48).

### 5.1 Strengths and limitations

There are several strengths of our study. We utilized a well-established implementation science framework to identify the determinants of implementing STEADI in outpatient physical therapy. We utilized mixed methods for data triangulation and contextual understanding of our findings. Our respondents included a diverse sample of key partners who would be impacted by STEADI implementation, enabling us to identify contextually specific determinants to optimize implementation. However, limitations include that our purposive samples of healthcare providers, patients, and care partners were from one state and healthcare system and their sociodemographics were homogenous. Participants self-selected into the study, and results rely on perception-based outcomes that may not fully capture actual practice behaviors. These limitations may hinder the transferability of our findings. Future studies would be beneficial to identify perceptions of STEADI integration in physical therapy among a more diverse sample of participants in a variety of locations.

## Data Availability

Data are available upon reasonable request from the corresponding author.
