# Supplementary material for: Implementing STEADI for routine falls prevention of all older adults attending outpatient physical therapy: key partner perspectives
Source: Front Health Serv. 2026 Feb 18;5:1718490. doi: 10.3389/frhs.2025.1718490 (PMC12977011; doi:10.3389/frhs.2025.1718490)
Supplement: Supplementary file 1 [file Table1.docx]

Supplementary Tables STEADI Barriers and Facilitators

Table S1. Therapy clinician reported measures used to *screen* an older adult’s fall risk who is attending outpatient therapy

(not including STEADI screening questionnaire, Timed up and Go, 30-second chair stand, or 4-stage balance test).

| Screening measure | *n(%)* |
| --- | --- |
| Lower Extremity Functional Scale (LEFS) | 11(68.8) |
| Activities Specific Balance Confidence Scale (ABC) | 10(62.5) |
| Oswestry Disability Index (ODI) | 5(31.3) |
| Dizziness Handicap Index (DHI) | 5(31.3) |
| Patient-Specific Functional Scale (PSFS) | 4(25.0) |
| Falls Efficacy Scale (FES) | 1(6.3) |
| AMPAC outpatient form | 0(0.0) |
| Other | 5(31.3) |
| Berg Balance Scale (BBS) | 2(12.5) |
| Tinetti Performance Oriented Mobility Assessment | 2(12.5) |
| Functional Gait Assessment (FGA) | 2(12.5) |
| Mini-best Test (miniBest) | 1(6.3) |
| Gait velocity | 1(6.3) |
| 2-minute walk test/6-minute walk test | 1(6.3) |
| 5-times sit to stand test | 1(6.3) |
| Dual-task TUG | 1(6.3) |
| Gans sensory organization performance test | 1(6.3) |
| Freezing of Gait Questionnaire (FOG) | 1(6.3) |
| I do not screen for fall risk | 1(6.3) |

Note*:* Participants could choose more than one test and enter more than one test in the other category.

Table S2. What functional/balance *assessments*/outcomes measures do you use to assess fall risk?

(not including Timed up and Go, 30-second chair stand, or 4-stage balance test).

| Measures | *n(%)* |
| --- | --- |
| Berg Balance Scale (BBS) | 12(75.0) |
| 5-times sit to stand | 10(62.5) |
| Mini best test | 8(50.0) |
| Tinetti POMA | 8(50.0) |
| 10-meter walk or gait speed | 8(50.0) |
| Dynamic Gait Assessment (DGI) | 7(43.8) |
| Functional Gait Assessment (FGA) | 7(43.8) |
| 6-minute walk | 6(37.5) |
| Other functional outcome measures | 4(25.0) |
| Functional Reach Test (FRT) | 2(12.5) |

Table S3. Reasons affecting therapists’ decision to use STEADI fall risk questionnaire, physical tests, assessments, and interventions

|  | ***Questionnaire*** | ***Physical tests*** | ***Assessments*** | ***Interventions*** |
| --- | --- | --- | --- | --- |
| Reasons | *n(%)* | *n(%)* | *n(%)* | *n(%)* |
| Time to complete | 8(50.0) | 10(62.5) | 15(93.8) | 12(75.0) |
| My choice to use other tests | 5(31.3) | 7(43.8) | 6(37.5) | 6(37.5) |
| Complexity | 3(18.8) | 3(18.8) | 5(31.3) | 6(37.5) |
| My employer recommendations | 6(37.5) | 2(12.5) | 3(18.8) | 3(18.8) |
| Not being familiar | 5(31.3) | 2(12.5) | 5(31.3) | 3(18.8) |
| Not appropriate for my patients | 2(12.5) | 4(25.0) | 2(12.5) | 2(12.5) |
| I do not see value | 0(0.0) | 0(0.0) | 0(0.0) | 0(0.0) |

Note: Respondents could check all that apply.

Table S4. Therapists - Stage of Change for Implementing STEADI

|  |  | | *Pre-survey* | *Post-survey* |
| --- | --- | --- | --- | --- |
| Stage of Change |  | | *n(%)* | *n(%)* |
|  |  |  |  |  |
| Contemplation | I am aware of it and am considering using it in the next 6 months. | | 9(56.3) | 12(75.0) |
| Pre-contemplation | I do not use it and I do not intend to use it in the foreseeable future. | | 5(31.3) | 1(6.3) |
| Preparation | I am ready to use it and will start using it in the next 30 days. | | 1(6.3) | 2(12.5) |
| Maintenance | I have been using it for more than 6 months and plan to continue to use it. | | 1(6.3) | 1(6.3) |

Table S5. Physician perceptions of appropriateness of physical therapists and physical therapist assistants conducting fall risk assessments and interventions

|  | | *n(%)* | | | | |
| --- | --- | --- | --- | --- | --- | --- |
| *Which of the following fall risk assessments do you feel are appropriate for physical therapists or physical therapist assistants to screen or assess for older adults who screen at risk of falls?* | | | | | | |
| Foot problems and footwear | | | 5(100.0) | | | |
| Home safety | | | 5(100.0) | | | |
| Vision screen/encourage yearly check | | | 5(100.0) | | | |
| Orthostatic hypertension | | | 4(80.0) | | | |
| Comorbidities (e.g. depression, osteoporosis) | | | 4(80.0) | | | |
| Medications that increase fall risk | | | 4(80.0) | | | |
| Vestibular function* | | | 4(80.0) | | | |
| Cognition function* | | | 3(60.0) | | | |
| Vitamin D intake | | | 3(60.0) | | | |
| Urinary incontinence | 2(40.0) | | | | | |
| *Which of the following interventions, if any, do you think are appropriate for a physical therapist or physical therapist assistant to do with an older adult who is at risk of falls and attending physical therapy for any reason?* | | | | | | |
| Balance training | | | 5(100.0) | | | |
| Strength training | | | 5(100.0) | | | |
| Endurance training | | | 5(100.0) | | | |
| Gait training (including using assistive devices properly) | | | 5(100.0) | | | |
| ADL training | | | 5(100.0) | | | |
| Activity modifications to increase safety | | | 5(100.0) | | | |
| Vestibular rehab | | | 5(100.0) | | | |
| Caregiver training | | | 5(100.0) | | | |
| Home safety recommendations | | | 5(100.0) | | | |
| Footwear modifications/recommendations | | | 5(100.0) | | | |
| Family/caregiver education | | | 5(100.0) | | | |
| Add supplemental vitamin D | | | 2(40.0) | | | |
| Manage orthostatic hypotension | | | 1(20.0) | | | |
| Modifications of medications | | | 0(0.0) | | | |
| *Which of the following recommendations if any, do you think are appropriate for a physical therapist or physical therapist assistant to suggest for an older adult follow-up with or recommend the primary care physician provide a referral to address modifiable fall risk factors among older adults who were identified as at risk of falls - regardless of the reason they are attending physical therapy?* | | | | | |  |
| Referral back to primary care provider for management of orthostatic hypotension | | | | | 5(100.0) |  |
| Referral back to primary care provider for management of a medical issue | | | | | 5(100.0) |  |
| Referral to primary care provider or pharmacist for medication management | | | | | 5(100.0) |  |
| Recommend the older adult see an optometrist for vision issues | | | | | 5(100.0) |  |
| Refer older adult to social worker or care coordinator for resources | | | | | 5(100.0) |  |
| Request the older adult be seen by another PT who specializes in a different  area of practice | | | | | 5(100.0) |  |
| Request the referring provider refer to Occupational therapy | | | | | 5(100.0) |  |
| Request the referring provider refer to Speech and language pathology  Request the referring provider refer to Home Health Services | | | | | 5(100.0) |  |
| Request the referring provider write a prescription for durable medical equipment  (assistive devices and ADL equipment) | | | | | 5(100.0) |  |
| Recommend the older adult attend a community exercise program | | | | | 5(100.0) |  |
| Referral back to primary care provider for management of orthostatic hypotension | | | | | 5(100.0) |  |
| Referral back to primary care provider for management of a medical issue | | | | | 5(100.0) |  |
| Referral to primary care provider or pharmacist for medication management | | | | | 5(100.0) |  |
| Recommend the older adult see an optometrist for vision issues | | | | | 5(100.0) |  |
| Refer older adult to social worker or care coordinator for resources | | | | | 5(100.0) |  |

*Fall risk factors not included in STEADI

Table S6. Patients/older adults’ perceived acceptability of fall risk management therapy and rehabilitation interventions

| Question | *n(%)* | |  |  |
| --- | --- | --- | --- | --- |
| *Which of the following do you feel are acceptable for a physical therapist to do as part of fall risk management while an older adult is attending outpatient physical therapy for any reason? Check all that apply.* | | |  |  |
| Ask questions verbally or on a questionnaire about fall risk | 10(100.0) | |  |  |
| Ask questions verbally or on a questionnaire about history of falls | 9(90.0) | |  |  |
| Check leg strength | 10(100.0) | |  |  |
| Check walking ability | 10(100.0) | |  |  |
| Check balance | 10(100.0) | |  |  |
| Tell me how to decrease my risk of falling | 9(90.0) | |  |  |
| Recommend I see another healthcare provider to decrease my risk of falling | 8(80.0) | |  |  |
| Check my vision (seeing far and near) | 6(60.0) | |  |  |
| Check my feet and shoes | 9(90.0) | |  |  |
| Check my blood pressure | 7(70.0) | |  |  |
| I do not feel it is appropriate for a physical therapist to include any of the above items for fall risk management while an older adult is being seen for another reason | 1(10.0) | |  |  |
| *Which of the following rehabilitation interventions do you feel are acceptable for a physical therapist to include in outpatient physical therapy for an older adult identified as being at risk of falling but who is attending physical therapy for any reason?* | | | | |
| Provide strengthening exercises | | 10(100.0) | |  |
| Improve balance with walking | | 10(100.0) | |  |
| Improve balance during standing activities | | 10(100.0) | |  |
| Provide and/or teach someone how to use a walking device | | 10(100.0) | |  |
| Provide and/or teach someone how to use equipment to improve safety | | 9(90.0) | |  |
| Recommend changes to the home to improve safety | | 8(80.0) | |  |
| Recommend types of shoes to decrease risk of falling | | 9(90.0) | |  |
| Recommend an older adult do a community exercise program such as Silver Sneakers or Tai Chi. | | 8(80.0) | |  |
| Refer an older adult to another healthcare provider to address fall risk factors related to medicine or medical issues. | | 8(80.0) | |  |
| I do not think it is appropriate for a physical therapist to do any of the above interventions for an older adult at risk of falling who is attending therapy for another reason. | | 0(0.0) | |  |

Table S7. Care partners’ perceived acceptability of fall risk management therapy and rehabilitation interventions

| Question | *n(%)* | |  |
| --- | --- | --- | --- |
| *Which of the following do you feel are acceptable for a physical therapist to do as part of fall risk management while an older adult is attending outpatient physical therapy for any reason? Check all that apply.* |  | |  |
| Ask questions verbally or on a questionnaire about fall risk | 10(100.0) | |  |
| Ask questions verbally or on a questionnaire about history of falls | 10(100.0) | |  |
| Check leg strength | 10(100.0) | |  |
| Check walking ability | 10(100.0) | |  |
| Check balance | 10(100.0) | |  |
| Tell me how to decrease my risk of falling | 10(100.0) | |  |
| Recommend I see another healthcare provider to decrease my risk of falling | 7(70.0) | |  |
| Check my vision (seeing far and near) | 7(70.0) | |  |
| Check my feet and shoes | 9(90.0) | |  |
| Check my blood pressure | 8(80.0) | |  |
| I do not feel it is appropriate for a physical therapist to include any of the above items for fall risk management while an older adult is being seen for another reason | 0(0.0) | |  |
| *Which of the following rehabilitation interventions do you feel are acceptable for a physical therapist to include in outpatient physical therapy for an older adult identified as being at risk of falling but who is attending physical therapy for any reason?* | |  | |
| Provide strengthening exercises | | 10(100.0) |  |
| Improve balance with walking | | 10(100.0) |  |
| Improve balance during standing activities | | 10(100.0) |  |
| Provide and/or teach someone how to use a walking device | | 10(100.0) |  |
| Provide and/or teach someone how to use equipment to improve safety | | 9(90.0) |  |
| Recommend changes to the home to improve safety | | 8(80.0) |  |
| Recommend types of shoes to decrease risk of falling | | 10(100.0) |  |
| Recommend an older adult do a community exercise program such as Silver Sneakers or Tai Chi. | | 9(90.0) |  |
| Refer an older adult to another healthcare provider to address fall risk factors related to medicine or medical issues. | | 7(70.0) |  |
| I do not think it is appropriate for a physical therapist to do any of the above interventions for an older adult at risk of falling who is attending therapy for another reason | | 0(0.0) |  |
