# Supplementary figures and images for: Implementing STEADI for routine falls prevention of all older adults attending outpatient physical therapy: key partner perspectives
Source: Front Health Serv. 2026 Feb 18;5:1718490. doi: 10.3389/frhs.2025.1718490 (PMC12977011; doi:10.3389/frhs.2025.1718490)

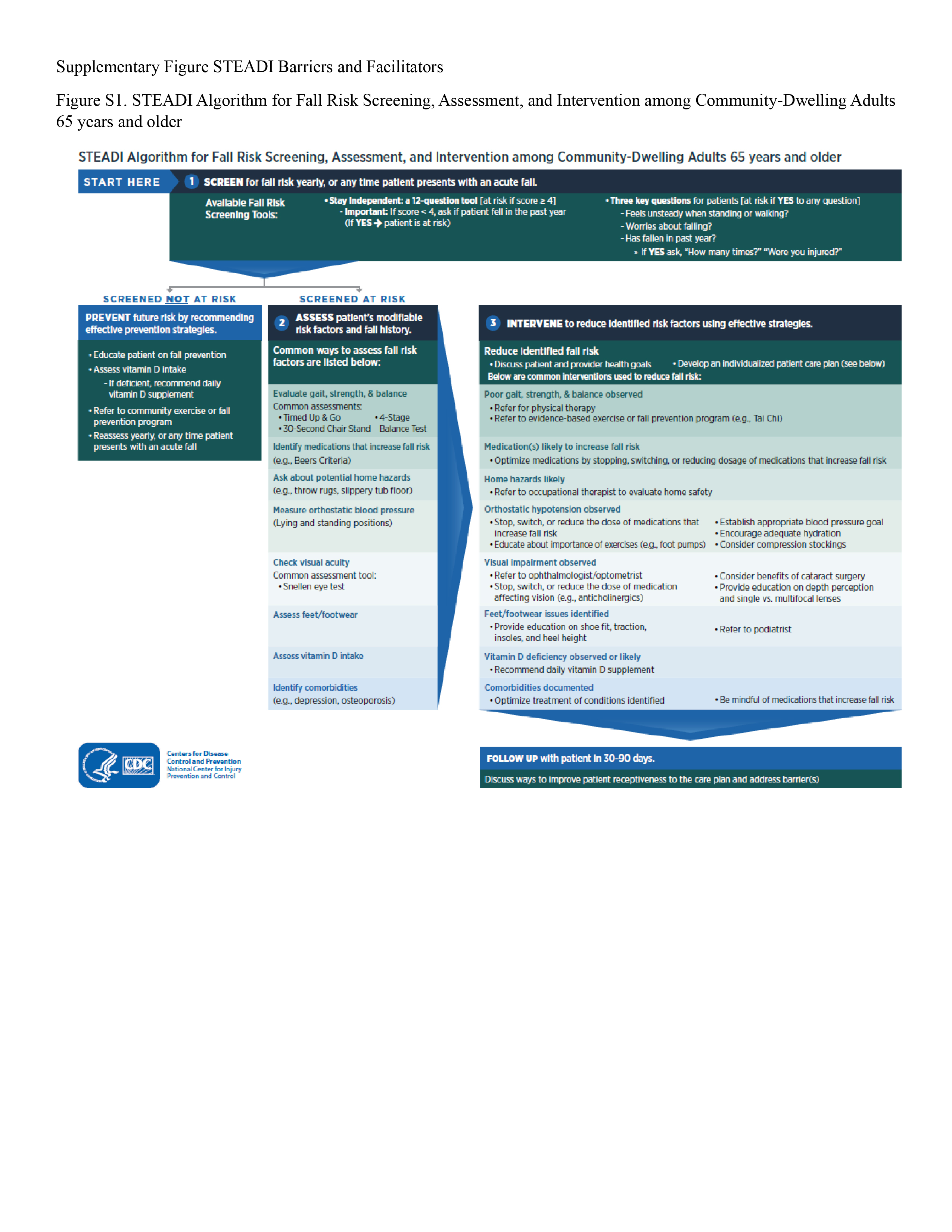

Supplement: Supplementary file 2 [file Image1.jpeg]
